# Spatial niche remodeling of senescent liver-resident immune cells and its role in chronic liver diseases

**DOI:** 10.3389/fmed.2026.1899423

**Published:** 2026-08-18

**Authors:** Fukang Mao, Keji Shan

**Affiliations:** The First Affiliated Hospital of Kunming Medical University, Kunming, Yunnan, China

**Keywords:** cellular senescence, chronic liver disease, Kupffer cells, liver-resident immune cells, senescence-associated secretory phenotype, single-cell transcriptomics, spatial niche remodeling, tissue-resident memory T cells

## Abstract

The liver serves the triple functions of metabolism, detoxification, and immune surveillance. Its unique immune microenvironment is shaped by continuous exposure to gut-derived antigens, pathogen-associated molecular patterns (PAMPs), and metabolites arriving via the portal vein, necessitating a delicate equilibrium between immune tolerance and effector activation. This equilibrium relies on the coordinated activities of diverse liver-resident immune cell populations—including Kupffer cells (KCs), liver sinusoidal endothelial cells (LSECs), hepatic stellate cells (HSCs), dendritic cells (DCs), tissue-resident memory T cells (TRM), innate-like T cells, including mucosal-associated invariant T (MAIT) cells, natural killer T (NKT) cells, and γδ T cells, innate lymphoid cells (ILCs, encompassing conventional NK cells and helper ILC subsets), and neutrophils. With advancing age and chronic injury, these resident immune cell populations undergo profound senescence-associated phenotypic reprogramming that is spatially organized along the portal-to-central axis of the hepatic lobule. Key mechanisms include: telomere dysfunction and DNA damage accumulation driving persistent activation of p53/p21 and p16/Rb pathways; mitochondrial dysfunction with mitochondrial DNA (mtDNA) leakage fueling the senescence-associated secretory phenotype (SASP) via the cyclic GMP-AMP synthase (cGAS)–stimulator of interferon genes (STING) pathway; epigenetic age acceleration, including genome-wide H3K27me3 heterochromatinization; and metabolic reprogramming toward glycolysis and lipid accumulation. This review proposes a “spatial niche remodeling” framework to integrate these cell-intrinsic senescence programs with their lobular context, intercellular communication network rewiring, and pathogenic roles across the spectrum of chronic liver disease—from steatosis through steatohepatitis, fibrosis, cirrhosis, to hepatocellular carcinoma. We critically evaluate emerging senotherapeutic strategies targeting specific liver-resident immune cell subsets, discuss the barriers to clinical translation, and identify priority areas for future investigation, including the application of spatial multi-omics, humanized models, and epigenetic clock-guided clinical trials.

## 1 Introduction

### 1.1 The liver as an immunological frontier

The liver is the only mammalian organ with a dual blood supply—approximately 75% of hepatic blood flow arrives via the portal vein, directly conveying gut-absorbed nutrients, microbial products, and xenobiotics, while the remaining 25% arrives via the hepatic artery (1). This unique anatomical configuration positions the liver at the frontline of immune surveillance, where it must simultaneously maintain tolerance toward harmless dietary antigens and commensal-derived molecules while retaining the capacity for rapid effector responses against pathogens (1, 2).

This immunological tightrope is orchestrated by a diverse repertoire of liver-resident immune cells that are precisely organized in space. Kupffer cells (KCs)—the largest population of tissue-resident macrophages in the body—adhere to the luminal side of hepatic sinusoids, continuously scanning passing blood for pathogens and debris (3, 4).

### 1.2 Global population aging and chronic liver disease

Population aging is advancing at an unprecedented pace. According to the World Health Organization, the global population aged 60 years and older will exceed 2 billion by 2050.

The impact of aging on the liver extends far beyond parenchymal hepatocytes. Arguably more profound alterations occur within the hepatic immune microenvironment, encompassing comprehensive remodeling of immune cell composition, phenotype, function, and spatial organization (5–7). These age-related immune alterations are particularly consequential in the liver, where chronic low-grade inflammation (“inflammaging”) intersects with disease-specific drivers such as lipotoxicity in metabolic dysfunction-associated steatotic liver disease (MASLD), viral persistence in chronic hepatitis, and gut-derived microbial translocation in cirrhosis.

### 1.3 Cellular senescence: beyond cell-autonomous arrest

Cellular senescence is a core hallmark of aging, defined as a state of irreversible proliferative arrest triggered by critically short telomeres, DNA damage, oxidative stress, oncogene activation, mitochondrial dysfunction, and other stressors (8, 9). Far from being a purely cell-autonomous phenomenon, senescence profoundly reshapes tissue microenvironments.

In the liver, SASP factors exert paracrine effects that propagate senescence to neighboring cells (secondary or paracrine senescence), activate quiescent HSCs into matrix-producing myofibroblasts, recruit inflammatory monocytes and neutrophils, and impair the effector functions of surveilling lymphocytes (10, 11). The concept that senescence is both a tumor-suppressive mechanism (via cell-cycle arrest) and a tumor-promoting process (via SASP-driven chronic inflammation and extracellular matrix (ECM) remodeling) is particularly relevant in the liver, where chronic inflammation is the dominant carcinogenic pathway (10).

### 1.4 Spatial niche remodeling: a conceptual framework

Along the portal-to-central axis of the hepatic lobule, gradients of oxygen tension (zone 1: ~60–65 mmHg, approximately 9–11% O₂; zone 3: ~30–35 mmHg, approximately 5–7% O₂), nutrient concentrations, Wnt/*β*-catenin morphogen activity, and gut-derived microbial product exposure create distinct microenvironments—or “immune niches”—that imprint region-specific phenotypes on resident immune cells (12, 13). This oxygen gradient arises from the unidirectional blood flow through the hepatic sinusoids, with progressive oxygen extraction by hepatocytes in zones 1 and 2, leaving zone 3 at the lowest oxygen tension and consequently most susceptible to ischemic and metabolic injury (13). Portal-zone macrophages express high levels of scavenger receptors (MARCO, CD163) and the anti-inflammatory cytokine IL-10, functioning as a tolerogenic barrier against gut-derived commensals (14).

With aging and chronic injury, these spatially organized immune niches undergo progressive remodeling—a process we define as spatial niche remodeling. This encompasses three interrelated dimensions: (1) cell-intrinsic senescence—differential susceptibility of immune cells in distinct lobular zones to senescence induction; (2) spatial redistribution—altered enrichment or depletion of specific immune subsets along the lobular axis; and (3) communication network rewiring—changes in the SASP secretome, chemokine gradients, and metabolic-immune crosstalk that alter the functional connectivity of the hepatic immune ecosystem. Importantly, sex differences represent an additional layer of complexity in spatial niche remodeling. Estrogen signaling via estrogen receptor-*α* (ERα) exerts anti-inflammatory and anti-fibrotic effects in the liver, while androgen signaling may promote pro-fibrotic responses. Premenopausal women exhibit lower rates of MASLD progression and hepatocellular carcinoma (HCC) incidence compared to age-matched men, a protection that is lost after menopause, implicating sex hormones in hepatic immune regulation. However, sex-stratified analyses of liver immune senescence are virtually absent from the current literature, representing a critical knowledge gap that we highlight throughout this review.

### 1.5 Technological breakthroughs enabling spatial dissection

Several recent technological advances have made it possible to investigate spatial niche remodeling at unprecedented resolution. Single-cell and single-nucleus RNA sequencing (scRNA-seq, snRNA-seq) have revealed remarkable immune cell heterogeneity, identifying dozens of novel liver immune cell subsets and transitional states (5, 15, 16). Spatial transcriptomics platforms (10x Visium, MERFISH, Slide-seq, Xenium) anchor these transcriptional states to precise anatomical coordinates within the hepatic lobule (12, 17, 18). However, several technical limitations warrant consideration when interpreting spatial data in the context of liver immune senescence. Current widely used spatial transcriptomics platforms (e.g., 10x Visium) operate at 55-μm spot resolution, which captures transcriptomes from multiple cells (5–15 cells per spot in liver tissue), precluding true single-cell spatial resolution and potentially averaging senescence signals across senescent and non-senescent cells within a spot. MERFISH and Xenium achieve subcellular resolution but rely on pre-designed gene panels (typically 300–500 genes) that may not comprehensively capture senescence-associated transcriptional programs. Moreover, gene capture efficiency varies spatially due to differences in tissue permeability, and dropout rates for lowly expressed genes (including many senescence markers such as p21/Cdkn1a) are high. Critically, spatial transcriptomic data demonstrate correlative patterns—co-localization of senescence markers with specific lobular zones—but cannot establish causality or distinguish between cell-intrinsic senescence programs and transcriptomic changes induced by the local microenvironment. Validation of spatial findings in human tissues remains challenging due to limited access to fresh, well-preserved human liver specimens spanning the full age spectrum and the spectrum of chronic liver disease. Emerging technologies including spatial proteomics (imaging mass cytometry, MIBI-TOF, CODEX) and spatial metabolomics are beginning to complement transcriptomic data but have not yet been systematically applied to liver immune senescence (Table 1).

**Table 1:** Key single-cell and spatial transcriptomic studies of liver immune aging and chronic liver disease.

Study	Technology	Species	Disease model/tissue	Key findings	Major limitations	Ref.
Liu et al. (2023)	scRNA-seq (89,542 cells, 9 subsets)	Mouse	Aged liver (4 ages); human transplant validation	Cxcl2 + senescence-like macrophage subset enriched in aged liver; CXCL2–CXCR2 neutrophil recruitment→NETosis→age-related injury	scRNA-seq: no spatial coordinates; low p21/Cdkn1a detection due to dropout; limited human validation; correlative, not causal	(5)
Duan et al. (2023)	Bulk/scRNA-seq + functional experiments	Mouse	Young/aged + diet MASH; human MASH biopsies	C-kit loss in pericentral LSECs→CXCR4 upregulation→aberrant LSEC–macrophage–neutrophil crosstalk; C-kit+ LSEC infusion reverses age-associated steatosis/NASH	Bulk + scRNA-seq: no single-cell spatial resolution; functional validation limited to murine models	(26)
Miyamoto et al. (2024)	10x Visium spatial + scRNA-seq + 2-photon imaging	Mouse	Healthy liver; commensal-driven inflammation model	MARCO+ immunosuppressive macrophages (MP2) enriched in periportal zone; IL-10-mediated protection against commensal-driven inflammation	10x Visium: 55-μm spot resolution (multicellular); correlative spatial patterns, not causal; no senescence-specific assessment	(14)
Andrews et al. (2024)	scRNA-seq + snRNA-seq + spatial transcriptomics	Human	Healthy & PSC liver	First comprehensive immune atlas of PSC liver; myeloid hyperactivation/exhaustion phenotypes; chronic cytokine markers in late-stage lesions	Disease-specific (PSC); limited aged samples; no senescence markers systematically assessed	(17)
Guilliams et al. (2022)	Spatial proteogenomics (CITE-seq)	Mouse & Human	Healthy liver (evolutionary conserved niches)	Evolutionarily conserved macrophage niches; KC zonation validated at proteomic level; niche signals conserved across 7 vertebrate species	CITE-seq: pre-designed protein panel (limited targets); no senescence-specific antibodies included	(70)
Ramachandran et al. (2019)	scRNA-seq (>100,000 cells)	Human	Cirrhotic liver	Fibrotic niche resolved at single-cell level; TREM2 + CD9 + scar-associated macrophages; novel mesenchymal subpopulations identified	scRNA-seq: enzymatic dissociation biases cell recovery; no spatial coordinates; no senescence markers assessed	(16)

scRNA-seq, single-cell RNA sequencing; snRNA-seq, single-nucleus RNA sequencing; MASH, metabolic dysfunction-associated steatohepatitis; LSEC, liver sinusoidal endothelial cell; PSC, primary sclerosing cholangitis; KC, Kupffer cell.

### 1.6 Scope of this review

This review aims to provide a comprehensive synthesis of current knowledge regarding senescent remodeling of liver-resident immune cells and their spatial niches in chronic liver disease. Throughout this review, we emphasize the translational gap between murine models—which provide the majority of mechanistic evidence—and human disease, and we critically evaluate the human validation data where available. Section 2 reviews the molecular hallmarks of liver cell senescence with emphasis on their spatial dimensions. Section 3 describes the spatial organization principles of the hepatic immune microenvironment. Section 4 provides cell-type-specific analyses of senescence programs and spatial niche remodeling across major liver-resident immune populations, with explicit attention to where in the lobule senescence occurs and how zonation matters functionally. Section 5 examines the remodeling of intercellular communication networks in the aging liver, focusing on spatially organized SASP gradients, extracellular vesicle-mediated signaling, and chemokine network reprogramming. Section 6 analyzes the pathogenic roles of senescent immune niches across the progressive stages of chronic liver disease—from steatosis through steatohepatitis, fibrosis, cirrhosis, to HCC. Section 7 critically evaluates emerging therapeutic strategies targeting senescent liver immune niches, with emphasis on the barriers to clinical translation. Section 8 synthesizes key conclusions, identifies critical knowledge gaps, and outlines a forward-looking vision for future research leveraging spatial multi-omics, organoid models, and epigenetic clock-guided clinical trials.

## 2 Molecular hallmarks of liver cell senescence

### 2.1 Telomere dysfunction and the DNA damage response

Penrice et al. (19) comprehensively reviewed the role of telomere dysfunction in chronic liver disease, highlighting how telomere attrition links cellular aging to hepatic pathology across the spectrum from steatosis to cirrhosis and HCC.

Persistent DNA damage response (DDR) foci—termed DNA-SCARS (DNA segments with chromatin alterations reinforcing senescence)—serve as nuclear signaling hubs that maintain the senescent state. DNA-SCARS activate NF-κB and C/EBPβ, the master transcriptional regulators of the SASP program (8, 10).

### 2.2 The telomere–mitochondria aging axis

Telomere dysfunction and mitochondrial deterioration are mechanistically coupled in a feedforward aging loop. Telomere damage signals through p53 to repress PGC1α and PGC1β—the master transcriptional co-activators of mitochondrial biogenesis—resulting in reduced mitochondrial mass, impaired oxidative phosphorylation (OXPHOS), and elevated reactive oxygen species (ROS) production (19).

### 2.3 Mitochondrial dysfunction, mtDNA release, and the cGAS-STING pathway

Mitochondrial outer membrane permeabilization (MOMP) and leakage of mitochondrial DNA (mtDNA) into the cytosol represent recently appreciated drivers of senescence. Cytosolic mtDNA, owing to its bacterial ancestry (unmethylated CpG motifs), functions as a damage-associated molecular pattern (DAMP) that is sensed by the cyclic GMP-AMP synthase (cGAS)–stimulator of interferon genes (STING) pathway (20).

Torres et al. (21) reviewed the synergistic roles of mitochondrial dysfunction and NLRP3 inflammasome activation in alcoholic and non-alcoholic steatohepatitis.

### 2.4 Epigenetic aging

Epigenetic alterations constitute one of the twelve hallmarks of aging and are mechanistically interconnected with other aging hallmarks in the context of liver immune senescence (8). Telomere attrition and genomic instability generate persistent DNA damage signals that redistribute silencing chromatin marks, including H3K27me3, away from subtelomeric regions and toward genic loci involved in immune function and metabolism (19). Mitochondrial dysfunction not only drives senescence through mtDNA/cGAS-STING signaling but also depletes the NAD + pool required for sirtuin (SIRT1, SIRT6) deacetylase activity, thereby linking metabolic aging to epigenetic dysregulation (8, 20). Loss of proteostasis, particularly impaired autophagy (ATG5 decline in aged KCs), prevents the degradation of damaged mitochondria (mitophagy) and aggregated SASP factors, creating a proteotoxic feedforward loop (22). Deregulated nutrient sensing via mTORC1 hyperactivation supports the bioenergetic demands of SASP production while simultaneously suppressing autophagy, exemplifying the antagonistic hallmarks of aging (8, 10). Stem cell exhaustion in the aged bone marrow biases hematopoiesis toward myeloid differentiation at the expense of lymphoid lineages, reducing the supply of naïve T cells and NK cell progenitors while increasing the availability of monocyte precursors that replace embryonic KCs in the aging liver niche (5, 22). These interconnected hallmarks collectively establish a self-reinforcing aging ecosystem in which cell-intrinsic damage, metabolic dysfunction, and immune dysregulation are inextricably linked.

DNA methylation-based epigenetic clocks enable quantitative assessment of biological aging in liver disease. A study utilizing the Levine PhenoAge clock found that epigenetic age acceleration was significantly greater in HCC tumor tissue compared to adjacent normal liver, and that accelerated epigenetic aging was negatively correlated with NK cell infiltration (23).

### 2.5 The senescence-associated secretory phenotype

#### 2.5.1 Composition and heterogeneity

The SASP is the principal mechanism through which senescent cells influence their microenvironment. Its composition is highly heterogeneous, depending on the senescence-inducing stimulus, cell type, and duration of senescence (8, 10).

#### 2.5.2 Transcriptional regulation

The SASP transcriptional program is governed by a core NF-κB–C/EBPβ regulatory complex, with additional inputs from p38MAPK, mTOR, cGAS-STING, Notch, and JAK/STAT signaling pathways (8, 10). NF-κB binding sites are enriched in the promoter regions of canonical SASP genes (IL6, IL8, CXCL1, CXCL2, CCL2, and MMP family members).

#### 2.5.3 Spatial gradient effects

The spatial range of SASP action is a critical determinant of its tissue-level impact. Paracrine senescence is most potent within a spatially restricted zone around each senescent cell, creating discrete “senescence fields” whose dimensions are shaped by local blood flow velocity, extracellular matrix binding, and lymphatic drainage—factors that vary substantially across lobular zones; the precise spatial range in the human hepatic lobule has not been experimentally determined. In the hepatic sinusoid, SASP factors are subject to convective transport by blood flow in the portal-to-central direction, generating concentration gradients of key mediators such as TGF-*β*, IL-6, and CXCL chemokines along the lobular axis (4, 10). The distance-dependent effects are particularly consequential in zone 3, where lower blood flow velocity permits greater local accumulation of SASP factors, potentially explaining the heightened susceptibility of pericentral regions to senescence propagation. Furthermore, lymphatic drainage from the space of Disse to portal tract lymphatics creates an additional route for SASP factor distribution, selectively transporting lower-molecular-weight mediators (e.g., IL-8, CCL2) toward portal regions while larger SASP components (e.g., MMP complexes, EVs) remain concentrated near their senescent cell of origin. This dual convection-diffusion system means that the spatial impact of a senescent cell depends critically on its lobular location: a senescent KC in zone 1 releases SASP factors into high-flow blood that rapidly distributes them across downstream zones, whereas a senescent cell in zone 3 generates a more locally concentrated SASP field due to reduced convective clearance. Computational models of hepatic sinusoidal flow suggest that SASP factors may form zone-dependent concentration gradients. However, direct experimental measurement of these gradients in human tissue remains technically challenging and represents a priority for future spatial transcriptomic and proteomic studies.

### 2.6 The senescence–immune clearance imbalance

Krizhanovsky et al. (24) provided the seminal demonstration that inducing senescence in activated HSCs limits liver fibrosis *in vivo*, establishing the concept that cellular senescence can exert anti-fibrotic functions through cell-cycle arrest and immune-mediated clearance.

## 3 Spatial organization principles of the hepatic immune microenvironment

### 3.1 Hepatic lobular zonation and immune niches

The basic functional unit of the liver is the hepatic lobule, a hexagonal structure organized around a central vein, with portal triads (containing portal venule, hepatic arteriole, and bile ductule) at the periphery. Along the portal–central axis, three metabolic zones are distinguished: zone 1 (periportal, high oxygen tension ~60–65 mmHg, abundant nutrients, relatively low Wnt/*β*-catenin signaling), zone 2 (mid-lobular, intermediate), and zone 3 (pericentral, low oxygen tension ~30–35 mmHg, high lipid and xenobiotic metabolism, high Wnt/*β*-catenin signaling) (12, 13). Wnt ligands (Wnt2, Wnt9b) and R-spondin 3 (Rspo3) are secreted predominantly by pericentral LSECs, establishing a portal-to-central Wnt morphogen gradient that is essential for maintaining hepatocyte metabolic zonation (13, 25, 26).

**Figure 1:**
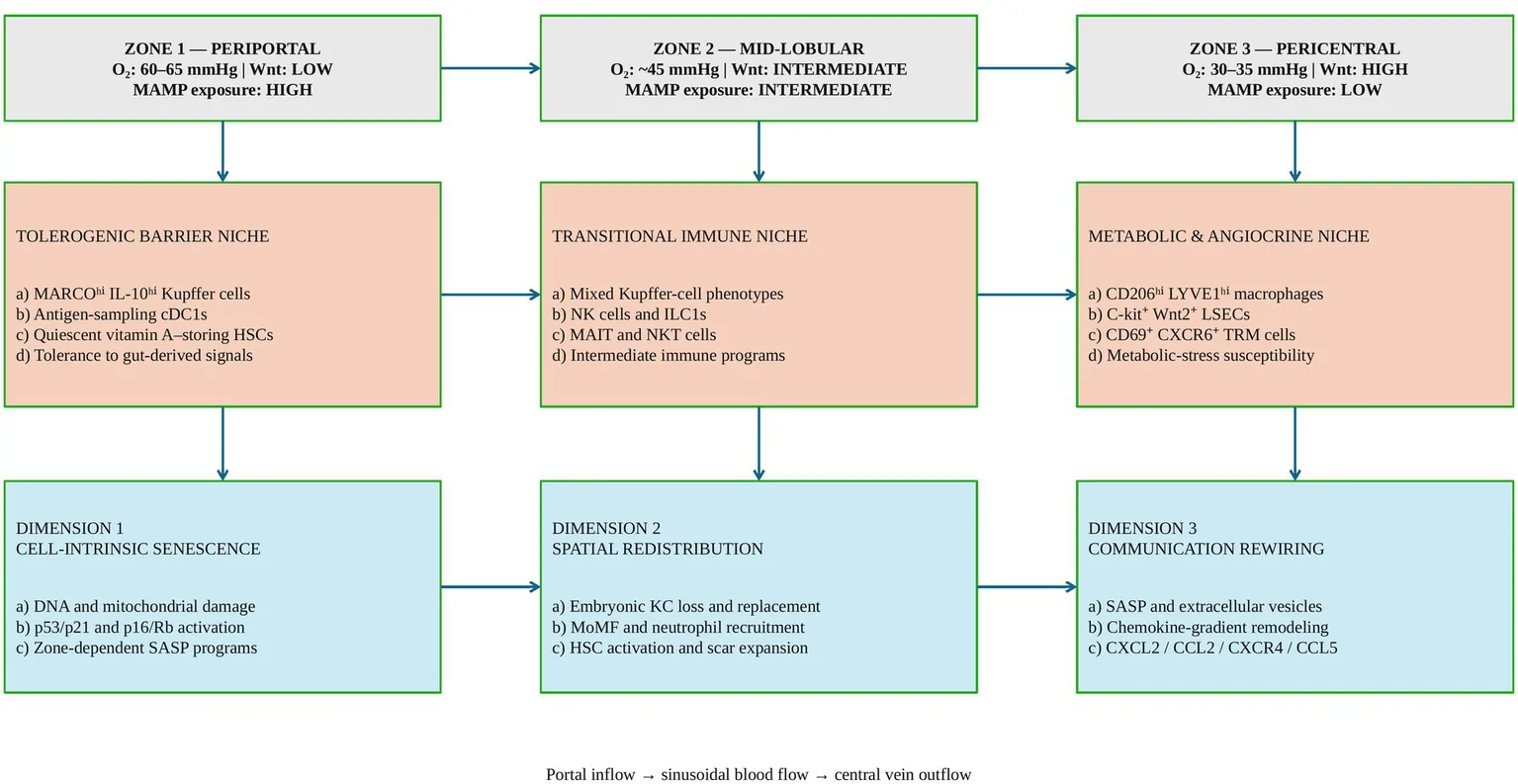
Hepatic lobular immune zonation and the three dimensions of spatial niche remodeling. The hepatic lobule is organized into three metabolic zones (periportal, mid-lobular, pericentral) defined by gradients of oxygen tension, Wnt/*β*-catenin morphogen activity, and gut-derived microbial product exposure. Each zone imprints distinct phenotypes on resident immune cells, summarized in the lower panels. Three dimensions of spatial niche remodeling are shown: Cell-intrinsic senescence, spatial redistribution of immune subsets, and communication network rewiring.

Periportal zone 1 KCs express high levels of MARCO, CD163, TIM4, Clec4F, TLR2, and the anti-inflammatory cytokine IL-10, equipping them with potent pathogen/internalization clearance and immunoregulatory capacity (4, 14). Miyamoto et al. (14) demonstrated that MARCO+ immunosuppressive macrophages are enriched in the periportal zone, where they provide IL-10-mediated protection against commensal-driven liver inflammation, establishing a critical spatial determinant of hepatic immune homeostasis. The spatial organization of these immune niches along the portal–central axis is schematically summarized in Figure 1.

LSECs exhibit parallel zonation: portal LSECs express higher levels of scavenger receptors (Stabilin-1/2, CD32b, CD36) and adhesion molecules (ICAM-1, VCAM-1), functioning as a filtration and immune cell docking interface; central LSECs express higher levels of Wnt2, Wnt9b, Rspo3, and C-kit, providing angiocrine signals that maintain zone 3 hepatocyte identity and metabolic function (25–27).

This zonation is actively maintained by microenvironmental signals: the Wnt/*β*-catenin morphogen gradient, established by Wnt2/Wnt9b/Rspo3 secretion from pericentral LSECs, shapes hepatocyte metabolic zonation with low activity in zone 1 and high activity in zone 3; gut-derived microbial products signal through TLRs and MYD88 on LSECs and KCs to maintain periportal immune phenotypes; bile acid gradients reinforce metabolic zonation; and the NOTCH pathway regulates LSEC fenestration and angiocrine output (4, 17, 26, 27).

### 3.2 The gut–liver axis as a spatial immune gradient generator

The gut–liver axis is a critical determinant of hepatic immune spatial organization. Portal venous blood delivers gut-derived microbial products, including lipopolysaccharide (LPS), peptidoglycan, flagellin, bacterial DNA, and metabolites including (short-chain fatty acids [SCFAs] and secondary bile acids) that impose a portal-to-central concentration gradient across the hepatic lobule (28–30).

Adhikary et al. (29) systematically reviewed how age-related gut dysbiosis drives chronic liver disease progression.

A breakthrough discovery by Leinwand et al. (31) demonstrated that the healthy liver harbors a low-biomass intrahepatic microbiome that dynamically programs NKT cell recruitment and activation via the CCL5-CCR5 axis, fundamentally reshaping our understanding of liver-commensal crosstalk and its age-dependent alterations.

## 4 Senescence and spatial niche remodeling across liver-resident immune cell populations

### 4.1 Kupffer cells and liver-resident macrophages

#### 4.1.1 Developmental origin, self-renewal, and spatial heterogeneity

KCs are the largest tissue-resident macrophage population in the body, originating during embryogenesis from yolk-sac-derived erythro-myeloid progenitors (EMPs) that seed the fetal liver by embryonic day 10.5, with additional contributions from fetal liver hematopoietic precursors during the perinatal period (3, 4, 32). Postnatally, KCs maintain themselves primarily through local self-renewal, independently of bone marrow-derived monocyte input under homeostatic conditions (32). The identity and maintenance of the KC pool critically depends on a specialized niche composed of LSECs (providing DLL4/Notch signals), HSCs (providing BMP9/10-ALK1-SMAD4 signals), and hepatocytes (providing lipid/desmosterol signals), which together induce the master transcription factor LXRα—a mechanism conserved across multiple vertebrate species (33).

The spatial heterogeneity of KCs has been comprehensively characterized (4, 14, 34). Periportal KCs (KC1) express a scavenger/immunoregulatory gene program (MARCO^hi^, CD163^hi^, Clec4F^hi^, IL-10^hi^), while pericentral KCs (KC2) express a metabolic gene program (CD206^hi^, LYVE1^hi^, TIM4^hi^, Hmox1^hi^, Cd36^hi^).

#### 4.1.2 Functional senescence of KCs

KCs undergo profound functional reprogramming with age, and this senescence is spatially heterogeneous. Liu et al. (5) performed scRNA-seq on mouse livers at four ages (newborn, suckling, young, aged), capturing 89,542 immune cells across 9 subsets—the most comprehensive single-cell atlas of liver immune aging to date. They identified a Cxcl2 + senescence-like macrophage subset that is enriched in aged livers and recruits neutrophils via CXCL2-CXCR2 signaling, driving NETosis and age-related liver injury. Critically, this senescent macrophage population is not uniformly distributed across the lobule; it is preferentially enriched in the periportal zone, where continuous exposure to gut-derived TLR ligands may synergize with cell-intrinsic aging programs to drive the senescence phenotype. Periportal KCs, owing to their high scavenger and TLR expression, may be particularly vulnerable to senescence induced by chronic low-level PAMP stimulation (“inflamma-aging”), whereas pericentral KCs may be more susceptible to metabolic and oxidative stress-driven senescence. This zone-specific vulnerability has direct functional implications: periportal KC senescence impairs the tolerogenic barrier, potentially permitting inappropriate immune activation against gut-derived antigens, while pericentral KC senescence may contribute to impaired lipid handling and steatosis progression.

Yao et al. (22) reviewed the multi-faceted impact of aging on the hepatic myeloid compartment: (1) hematopoietic stem cell myeloid skewing—increased common myeloid progenitors, decreased common lymphoid progenitors—leads to increased monocyte numbers in bone marrow and peripheral blood, secreting more TNF and IL-6; (2) KC numbers gradually increase with age, but their phagocytic capacity declines due to reduced ATG5-dependent autophagy, which promotes M1-like inflammatory polarization (↑IL-1β, TNF-*α*; ↓efferocytosis, ↓phagocytosis of pathogens and apoptotic debris); and (3) in aged rats, both M1-and M2-like KCs increase, reflecting the state of chronic low-grade immune activation (inflammaging) (6, 22).

#### 4.1.3 The SIRT1–NLRP3–fibrosis axis in aged KCs

Adjei-Mosi et al. (35) reported a landmark study in Aging Cell demonstrating that age-dependent loss of hepatic SIRT1 enhances NLRP3 inflammasome signaling and impairs liver fibrosis resolution. Aged mice developed more severe and persistent liver fibrosis compared to young mice following hepatotoxin-induced injury, with impaired fibrosis resolution even after cessation of the injurious stimulus.

Complementing these findings, Kaufmann et al. (36) demonstrated that myeloid cell-specific (LysM-Cre) deletion of NLRP3 markedly reduced liver inflammation and fibrosis in murine steatohepatitis, directly implicating KC/macrophage NLRP3 in metabolic liver disease.

#### 4.1.4 TREM2 + lipid-associated and scar-associated macrophages

In metabolic liver disease, a distinctive TREM2 + macrophage population emerges that has been variously termed lipid-associated macrophages (LAMs), scar-associated macrophages (SAMs), or NASH-associated macrophages (NAMs). These cells express high levels of TREM2, GPNMB, CD9, SPP1, and FABP5, and are enriched for endocytic, lysosomal, major histocompatibility complex class II (MHC-II) antigen presentation, and ECM remodeling gene programs (4, 37–39).

Hendrikx et al. (37) demonstrated that soluble TREM2 (sTREM2) plasma levels reflect the recruitment and expansion of TREM2 + macrophages that localize to fibrotic areas and limit NASH progression. sTREM2 has been validated as a non-invasive MASH biomarker: sTREM2 ≤ 38 ng/mL rules out MASH (sensitivity 90%, specificity 52%); sTREM2 ≥ 65 ng/mL rules in MASH (specificity 89%, sensitivity 54%) (38).

A critical molecular switch was identified in advanced MASH: the disintegrin metalloproteinase ADAM17 is upregulated, leading to excessive cleavage of TREM2’s ectodomain. This generates elevated plasma sTREM2 levels while simultaneously reducing membrane-bound TREM2 signaling, thereby crippling LAM protective functions (38).

Spatially, LAMs are predominantly enriched in pericentral zone 3, surrounding lipid-laden hepatocytes and hepatic crown-like structures, while SAMs localize to bridging fibrotic septa (portal–portal and portal–central) in close proximity to activated HSCs (4, 34). Lee et al. (39) demonstrated that these TREM2 + macrophages express matrix metalloproteinases (MMPs) to control fibrotic scar formation and promote fibrosis resolution.

#### 4.1.5 Macrophage niche replacement in chronic injury

In progressive chronic liver injury, the embryonic KC pool is progressively replaced by monocyte-derived macrophages (MoMFs) recruited via the CCL2–CCR2 axis (4, 22). This “niche occupancy” represents a fundamental restructuring of the hepatic macrophage landscape—Tim4 + Clec4F + embryonic KCs are lost (exhibiting a senescence-like loss of phagocytic capacity), and the macrophage pool is dominated by MoMFs that adopt partial KC-like phenotypes but differ functionally (4, 6).

### 4.2 Liver sinusoidal endothelial cells

#### 4.2.1 LSEC physiology and heterogeneity

LSECs form the wall of the hepatic sinusoid and are distinguished from all other vascular endothelia by the presence of fenestrae—transcellular pores of 50–200 nm diameter organized into sieve plates—and the absence of a continuous basement membrane. These features confer exceptionally high permeability, allowing direct contact between sinusoidal blood and hepatocyte microvilli in the space of Disse (25, 26).

McConnell et al. (25) provided a comprehensive review of four broad advances in LSEC biology: (1) LSEC heterogeneity—scRNA-seq-defined subpopulations with distinct portal versus central zonation patterns; (2) LSEC capillarization as the hallmark of sinusoidal dysfunction in cirrhosis and aging; (3) the spectrum of LSEC-derived angiocrine signals (Wnt, BMP, hepatocyte growth factor [HGF], CXCL chemokines) that maintain hepatocyte zonation, regulate HSC quiescence, and orchestrate immune cell recruitment; and (4) emerging therapeutic strategies targeting LSEC dysfunction, including statins, FXRa agonists, and C-kit restoration.

A detailed 2024 review of angiocrine signaling in the Journal of Hepatology outlined the LSEC secretome and its role in sinusoidal homeostasis (27). LSECs are the major source of CXCL chemokines (CXCL1, 2, 5, 8 for neutrophil recruitment) and CCL2 (for monocyte recruitment, via BRD4/NF-κB).

#### 4.2.2 LSEC senescence: capillarization and C-kit deficiency

The hallmark of LSEC senescence is capillarization—the progressive loss of fenestrae, formation of a subendothelial basement membrane, and phenotypic dedifferentiation toward a continuous capillary-type endothelium (25, 26). Capillarized LSECs show reduced nitric oxide (NO) production, via endothelial nitric oxide synthase (eNOS) downregulation, diminished Wnt/BMP angiocrine output, and a switch to a pro-inflammatory, pro-fibrotic, pro-thrombotic secretome (increased TGF-*β*, PDGF, Endothelin-1, and CXCL chemokines) (25, 27, 40).

Duan et al. (26) published a landmark study in Nature Aging that identified a specific molecular mechanism by which LSEC senescence drives steatohepatitis.

Kit gene deletion in mice worsened hepatic steatosis and exacerbated NASH-associated fibrosis and inflammation. Crucially, blocking CXCR4 signaling (with AMD3100/Plerixafor) abolished the LSEC–macrophage–neutrophil crosstalk and rescued the NASH phenotype in C-kit-deficient mice.

Abdulmajeed et al. (40) further emphasized the immunoregulatory role of LSECs in fatty liver disease, framing LSEC capillarization as a critical switch from homeostatic immune regulation (inhibiting disproportionate immune activation) to pro-inflammatory, pro-fibrotic signaling (via TGF-*β*-mediated HSC activation).

#### 4.2.3 Spatially heterogeneous LSEC senescence

LSEC senescence is spatially non-uniform. Pericentral (zone 3) LSECs are the first to exhibit C-kit downregulation, telomere shortening, and fenestrae loss during aging and MASH progression (26).

### 4.3 Hepatic stellate cells

#### 4.3.1 HSC physiology and immune functions

HSCs reside in the space of Disse and constitute approximately 5–8% of total liver cells. In their quiescent state, HSCs store vitamin A (retinyl esters) in characteristic lipid droplets—containing >80% of total body vitamin A—and maintain the homeostatic ECM of the space of Disse (basement membrane-like matrix composed primarily of collagen types IV and VI, laminin, and heparan sulfate proteoglycans) (41, 42).

Beyond their fibrogenic role, HSCs possess significant immunoregulatory capacity. Activated HSCs express MHC-II and co-stimulatory molecules (CD80, CD86), enabling them to function as non-professional antigen-presenting cells that can prime CD4 + T cell responses or induce T-cell anergy depending on context (43).

#### 4.3.2 The HSC senescence paradox

HSC senescence presents one of the most complex and therapeutically significant paradoxes in liver aging research—the “Jekyll-and-Hyde” nature of HSC senescence in fibrosis (10, 41). Critically evaluating which context predominates in human disease remains challenging: most evidence for the anti-fibrotic face of HSC senescence comes from murine models employing acute hepatotoxin-induced fibrosis (CCl₄) that resolve upon cessation of injury, whereas human chronic liver disease is characterized by persistent, multi-decade injury (viral, metabolic, or alcoholic) where senescence induction may be incomplete or of altered quality. In human cirrhotic livers, p53/p21-positive senescent HSCs are detectable but are vastly outnumbered by activated, proliferative myofibroblasts, suggesting that the balance under chronic injury conditions favors the pro-fibrotic face. Furthermore, the translational challenge is compounded by the fact that the same SASP factors that promote fibrosis (TGF-*β*, IL-6, CCL2) are also required for efficient immune-mediated clearance of senescent HSCs—thus, broad SASP suppression may paradoxically impair senescent cell removal. The periportal versus pericentral context further modulates this paradox: periportal HSCs, continuously exposed to gut microbial products, maintain a more pro-inflammatory SASP profile that may sustain fibrosis, whereas pericentral senescent HSCs, in a lower-inflammatory milieu, may adopt a more favorable, clearance-competent phenotype.

The pro-fibrotic face: Senescent hepatocytes and KCs release SASP factors (IL-6, TGF-*β*, CCL2, IL-1β) that paracrine-activate quiescent HSCs, driving their trans-differentiation into fibrogenic myofibroblasts (10, 44). Wijayasiri et al. (44) demonstrated that conditioned medium from senescent hepatocytes directly upregulates *α*-SMA and collagen I expression in HSCs in culture.

The anti-fibrotic face: However, inducing senescence specifically in activated HSCs can paradoxically limit fibrosis. Zhang et al. (41) reviewed the characteristics and mechanisms of HSC senescence.

SASP quality determines net effect: The critical variable is the “quality” of the SASP produced by senescent HSCs. A 2022 study in Acta Pharmaceutica Sinica B revealed that inhibition of ASCT2 (alanine-serine-cysteine transporter 2, a glutamine transporter) induces HSC senescence while simultaneously restricting the pro-inflammatory SASP through intervention in the IL-1α/NF-κB feedback loop (45). ASCT2 inhibition prevents glutaminolysis-dependent pro-inflammatory SASP production by binding to precursor IL-1α at Lys82, interfering with its maturation and secretion.

#### 4.3.3 NK cell immune surveillance of senescent HSCs

The timely clearance of senescent HSCs is a prerequisite for fibrosis resolution, and this task is primarily executed by NK cells. Zhang et al. (46) comprehensively reviewed the molecular crosstalk between NK cells and HSCs in liver fibrosis, detailing the receptor-ligand pairs (NKG2D–NKG2DL, NKp30–B7-H6, NKp44–NKp44L, and TRAIL–DR5) that mediate recognition and killing of activated and senescent HSCs, and how these interactions are disrupted in the fibrotic microenvironment.

Tao et al. (47) elucidated a precise molecular mechanism governing NK cell adhesion to HSCs, published in J Exp Med. They found that the prostaglandin E2 receptor EP3 is downregulated on NK cells from fibrotic mice and cirrhotic patients.

Immune evasion by HSCs: In advanced fibrosis and cirrhosis, however, HSCs deploy multiple strategies to evade NK cell killing (46): (1) TGF-*β* (produced abundantly by activated HSCs) downregulates NKG2D, 2B4, and NKp30 on NK cells; (2) HSCs can internalize NK cells via Rac1/Cdc42-dependent emperipolesis, leading to NK cell death within HSC endosomes; (3) senescent HSCs upregulate CD47, inhibiting macrophage efferocytosis; (4) some senescent HSCs shed NKG2D ligands via MMP-mediated ectodomain cleavage, rendering them invisible to NK cells; and (5) ECM stiffness alters NK–HSC contact dynamics.

#### 4.3.4 Spatial heterogeneity of HSC senescence

In the healthy lobule, periportal HSCs are predominantly involved in ECM homeostasis, whereas pericentral HSCs are more enriched in lipid metabolism and retinoid signaling pathways (12, 41). During chronic injury, HSC activation initiates in the periportal zone and progressively extends toward the pericentral zone, forming portal–portal and portal–central bridging fibrosis (4).

### 4.4 Liver-resident lymphocytes

#### 4.4.1 Tissue-resident memory T cells

Liver TRM cells have emerged as central players in hepatic immunity. Phenotypically characterized as CD69 + (inhibiting S1PR1-mediated tissue egress), CD103+/− (binding E-cadherin for intraepithelial retention), CXCR3+, and CXCR6 + (sinusoidal retention signals), TRM cells constitute a substantial fraction of the liver T-cell compartment (48, 49).

Koda et al. (49) demonstrated that CD8 + TRM cells promote liver fibrosis resolution by inducing apoptosis of activated HSCs through FasL–Fas interactions, revealing a protective function of TRM cells that may be compromised during aging.

Pallett et al. (50) reported an unexpected discovery in Nature: in cirrhotic patients, a subset of liver CD8 + TRM cells acquires CD14 (the LPS receptor) through membrane transfer from myeloid cells (monocyte/macrophage trogocytosis).

TRM cell aging: a critical knowledge gap. Systematic studies of age-related changes in liver TRM cells are virtually absent from the literature.

#### 4.4.2 Innate-like T cells: MAIT, NKT, and γδ T cells

Innate-like T cells (ILTCs) are disproportionately enriched in the human liver compared to peripheral blood, reflecting the liver’s status as an innate immune sentinel organ (51, 52). Yang et al. (52) provided a comprehensive review of innate-like T cells in liver disease, detailing how MAIT, NKT, and γδ T cells differentially respond to microbial metabolites, lipid antigens, and stress signals in the hepatic microenvironment.

MAIT cells constitute up to 20–40% of total human liver T cells (vs. 1–5% in peripheral blood), making them the most abundant T-cell subset in the liver (51, 52). MAIT cells recognize vitamin B2/B9 precursor metabolites (5-OP-RU and 5-OE-RU) presented by the non-classical MHC-I molecule MR1, thereby sensing microbial metabolic activity.

NKT cells recognize glycolipid antigens presented by CD1d. Liver NKT cells (predominantly invariant NKT/iNKT, with the semi-invariant Vα14-Jα18 TCR in mice and Vα24-Jα18 in humans) continuously patrol the sinusoids, recognizing CD1d + hepatocytes and KCs presenting endogenous and microbial glycolipids, and rapidly releasing large quantities of IFN-*γ* and IL-4 (31, 52).

γδ T cells constitute approximately 3–8% of human liver T cells, with a predominance of Vδ1 + cells (unlike peripheral blood, which is Vδ2 + −dominant) (51, 52). γδ T cells recognize stress-induced self-ligands (MICA/B, ULBPs), phosphoantigens, and lipid antigens in an MHC-independent manner.

Ibidapo-Obe et al. (53) focused on ILTC alterations in advanced chronic liver disease (cirrhosis).

#### 4.4.3 Innate lymphoid cells and natural killer cells

Innate lymphoid cells (ILCs) are a family of lymphocytes that lack somatically rearranged antigen receptors and are categorized into three major groups based on their transcription factor requirements and cytokine profiles: ILC1 (including conventional NK cells), ILC2, and ILC3—all of which are present in the liver (54). NK cells, the prototypical cytotoxic ILCs, are the core innate cytotoxic effectors in the liver and represent the dominant ILC subset. Their role in eliminating senescent HSCs via NKG2D-ligand interactions has been detailed in Section 4.3.3. Aged liver NK cells exhibit an “inflammatory shift”—increased IFN-*γ* production (sustaining the inflammatory milieu) but decreased CD107a degranulation and target cell killing efficiency, reflecting a functional dichotomy that impairs senescent cell clearance while promoting chronic inflammation (5, 6, 46). In cirrhosis, TGF-*β*-mediated downregulation of NKG2D and 2B4 cripples NK anti-fibrotic function, and HSC emperipolesis—a process whereby HSCs internalize and destroy NK cells via Rac1/Cdc42-dependent mechanisms—further depletes the functional NK cell pool (46). NK cell-derived exosomes (NK-Exos) containing miR-223 suppress HSC activation and liver fibrosis, representing a paracrine anti-fibrotic mechanism independent of direct cell contact (55).

Beyond conventional NK cells, the liver harbors tissue-resident ILC1s that are Eomes-independent and produce abundant IFN-*γ* and TNF-*α*, serving as the first line of antiviral and antitumor defense (6, 54). While cNK cells are primarily cytotoxic, tissue-resident ILC1s exhibit a more cytokine-dominant phenotype, and the balance between these subsets may be altered during aging. Importantly, the spatial distribution of liver ILC1s and NK cells is non-uniform: both populations patrol the sinusoidal lumen and are enriched at septal edges where senescent HSCs accumulate, positioning them for immune surveillance of the fibrotic niche.

ILC2: Hepatic ILC2s are activated by IL-33 (predominantly released by LSECs and HSCs upon injury) and secrete large amounts of IL-13 and IL-4. IL-13 activates HSCs via STAT6 signaling, promoting tissue remodeling and fibrosis (54).

ILC3: ILC3-derived IL-22 exerts a dual role in the liver (54): in acute injury, IL-22 promotes hepatocyte proliferation and survival via STAT3 signaling (protective); in chronic inflammation, sustained IL-22 can promote HSC activation. Critically, IL-22 also induces HSC senescence through STAT3/SOCS3-mediated activation of p53 and p21—this represents a key anti-fibrotic mechanism of ILC3s (54).

### 4.5 Neutrophils and NETosis

Neutrophil contributions to liver immune aging have gained substantial recent attention, particularly the role of NETosis in liver fibrosis.

Liu et al. (5) demonstrated that Cxcl2 + senescence-like macrophages in aged livers recruit neutrophils via CXCL2–CXCR2, and that NETosis is markedly enhanced in aged livers.

Fa et al. (56) provided a comprehensive review of NETosis in NAFLD: in simple steatosis, free fatty acids (linoleic acid, palmitic acid) induce NETosis via ERK/JNK/AKT pathways; in NASH, NETs recruit monocyte-derived macrophages and promote Treg differentiation via TLR4-mediated OXPHOS metabolic reprogramming; in the fibrosis stage, MPO-dependent NETosis drives HSC activation, and S1P/S1PR2 signaling promotes NET formation and fibrogenesis; in cirrhosis, NET markers (MPO-DNA complexes, H3Cit, NE) are markedly elevated in decompensated disease and correlate with portal vein thrombosis (56).

Maretti-Mira et al. (57) performed transcriptomic analysis of circulating neutrophils from MASH patients, revealing a dynamic shift with disease progression: in mild MASH (NAS ≤ 3, F0–1), mature neutrophils predominate with strong upregulation of NETosis genes (ELANE, MPO, AZU1) and pro-fibrotic factors (TGFB1, COL6A3); in advanced MASH (NAS ≥ 5, F3+), there is premature release of immature neutrophils (↑CD79B, CD38, CXCR4; ↓FCGR3A, CD10) with a distinct pro-inflammatory cytokine profile (TGFB3, LTBP4, IL34) (57).

Huang et al. (58) reviewed novel neutrophil subsets (CD177 + neutrophils, low-density neutrophils/LDNs) and their therapeutic target potential in non-cancer liver diseases.

Immunosenescence of neutrophils: a notable gap. Studies directly exploring age-related changes in hepatic neutrophil function are lacking. However, extrapolating from systemic neutrophil aging studies, several age-related alterations may be anticipated: aged neutrophils exhibit impaired chemotactic accuracy (reduced directional migration toward CXCL1/CXCL2 gradients), delayed apoptosis (contributing to prolonged inflammatory infiltration), enhanced NETosis propensity (driven by mitochondrial ROS and sustained calcium signaling), and altered metabolic programming with increased glycolysis. In the liver specifically, age-related changes in the CXCL2-CXCR2 and S1P-S1PR2 axes, combined with the emergence of immature neutrophil subsets (as documented by Maretti-Mira et al. in MASH patients), likely create a self-amplifying loop of neutrophil-mediated tissue injury. The spatial dimension is particularly understudied: neutrophils are recruited to distinct lobular zones depending on the chemokine source (periportal for CXCL2 from senescent KCs, pericentral for CXCR4 ligands from C-kit-deficient LSECs), and whether zone-specific neutrophil effector functions differ remains entirely unexplored.

### 4.6 B lymphocytes and tertiary lymphoid structures

B cells have received relatively little attention in liver immune aging research, but recent advances suggest their importance.

Hepatic B cell subsets: In the healthy liver, B cells constitute a minor fraction of lymphocytes (~5–10%). In chronic liver disease, B cell infiltration increases substantially.

Tertiary lymphoid structures (TLS) and liver fibrosis: TLS are ectopic lymphoid aggregates that form in chronically inflamed tissues, typically containing B-cell follicles (with germinal centers), T-cell zones, follicular dendritic cell (FDC) networks, and high endothelial venules (HEVs) (59). A 2022 Nature Communications study demonstrated that loss of canonical NOTCH signaling in vascular endothelial cells (Rbpj deletion) induces spontaneous TLS formation in the liver, kidney, and lung (59).

A 2025 Liver International study by Chu et al. reported that tertiary lymphoid structures (TLS) were present in 61.3% (46/75) of liver biopsies from patients with primary biliary cholangitis (PBC), with 26 patients harboring mature TLS (containing germinal centers with GL7 + IgD + B cells and CXCL13 + follicular dendritic cell networks) and 20 patients showing immature TLS. Mature TLS correlated with significantly higher serum biochemical indicators, elevated immunoglobulin levels, and a higher proportion of cirrhosis, suggesting that TLS maturation rather than mere presence drives disease progression (60). FTY720 (fingolimod, an S1P receptor modulator) inhibited TLS formation and relieved cholangitis and fibrosis in the dnTGFβRII mouse model of PBC, identifying S1P signaling as a potential therapeutic target for TLS-associated liver fibrosis (60).

In cirrhosis/acute-on-chronic liver failure (ACLF), scRNA-seq revealed alterations in B cell frequencies and distinct immunometabolic signatures in circulating immune cells (61).

Aging and hepatic B cells/TLS: The sustained chronic antigenic stimulation from gut microbial translocation and chronic SASP signals (particularly IL-6 and CCL chemokines) in the aged, cirrhotic liver may create conditions permissive for TLS formation. However, direct studies linking aging, hepatic B-cell biology, and TLS formation are virtually nonexistent.

### 4.7 Dendritic cells and aging

Hepatic DCs play a critical role in maintaining the balance between liver immune tolerance and activation. Major liver DC subsets include conventional DC1 (cDC1, specialized in cross-presentation, CD141+/XCR1+), conventional DC2 (cDC2, CD1c+/SIRP*α*+), and plasmacytoid DCs (pDCs, major producers of IFN-α/*β*) (6, 62).

In cirrhosis, hepatic DC antigen-presenting function is impaired—MHC-II and co-stimulatory molecule (CD80/CD86) expression is reduced, IL-12 production is diminished, and there is a shift toward promoting Treg differentiation (via IL-10, TGF-β, and PD-L1 expression), resulting in failed T-cell priming and immune tolerance (62). The spatial context of DC dysfunction is important: cDC1s are enriched in the periportal region where they sample portal blood-borne antigens and cross-present to CD8 + T cells, while cDC2s are more broadly distributed and specialize in CD4 + T cell priming. Age-related impairment of periportal cDC1 cross-presentation may be particularly consequential for anti-viral and anti-tumor immunity. Studies of aged liver DCs are extremely limited, but general principles of DC immunosenescence suggest: reduced type I interferon and IL-12 production after TLR stimulation, diminished antigen cross-presentation efficiency (due to reduced phagosomal ROS and altered endosomal pH), impaired migratory capacity toward CCR7 ligands (CCL19/CCL21), and a transcriptional shift toward tolerogenic programs (6, 62). In the aged liver specifically, the chronic SASP milieu (elevated IL-6, IL-10, TGF-*β*) may lock hepatic DCs into a semi-mature, tolerogenic state that fails to prime effective adaptive immunity while actively promoting Treg expansion, thereby contributing to the global decline in hepatic immune surveillance with age. Single-cell analyses of hepatic DC subsets across the human lifespan are urgently needed to define the trajectory and mechanisms of DC aging in the liver (Table 2).

**Table 2:** Overview of liver-resident immune cell senescence phenotypes and spatial niche characteristics.

Cell type	Lobular zone	Senescence markers	Functional changes	Ref.
Kupffer cells	Periportal (MARCO^hi^, IL-10^hi^); Pericentral (CD206^hi^, LYVE1^hi^)	↑p16/p21; ↓ATG5, SIRT1; ↑NLRP3, IL-1β; Cxcl2^+^ SASP-like subset	↓Phagocytosis/efferocytosis; M1 polarization; NLRP3 activation; CXCL2-mediated neutrophil recruitment	(4, 5, 35)
TREM2^+^ LAMs/SAMs	LAMs: pericentral (lipid); SAMs: fibrotic septa	↑TREM2, GPNMB, CD9, SPP1; ADAM17-mediated TREM2 shedding	Efferocytosis (protective); MMP-mediated ECM remodeling; ADAM17 cleavage→protection loss in advanced MASH	(37–39)
LSECs	Zone 3 (earliest senescence); progressive portal spread	↓C-kit; ↑CXCR4; ↓eNOS/NO; fenestrae loss (capillarization)	↓Wnt/BMP angiocrine signals; aberrant CXCL chemokines; impaired KC maintenance; ↑HSC activation	(25–27)
HSCs	Periportal (early activation); septal edges (senescent)	↑p53/p21, p16/Rb; ↑NKG2D ligands; ↑CD47; variable SASP	Proliferative arrest (anti-fibrotic) vs. SASP paracrine activation (pro-fibrotic); NK-mediated clearance	(24, 41, 46)
CD8^+^ TRM	Sinusoids; portal infiltrates; ascites	CD69^+^CD103^+^/^−^CXCR6^+^; ↑PD-1 (putative); CD14 acquisition in cirrhosis	Bystander activation in NASH; FasL-mediated HSC apoptosis; LPS sensing via CD14 in SBP	(48–50, 66)
MAIT cells	Sinusoidal/periportal (≤40% liver T cells)	↑PD-1, TIM-3 (exhaustion); MR1-dependent metabolite sensing	↓IFN-γ/granzyme B in HBV/HCV; IL-17A production in MASLD; functional decline in HCC	(51, 52)
NK cells	Sinusoidal lumen; septal edges	↓NKG2D, 2B4 (TGF-β); ↓EP3; ↑IFN-*γ* but ↓CD107a	↓Itga4–VCAM1 adhesion to HSCs; impaired senescent HSC clearance; NK-Exos (miR-223) anti-fibrotic	(46, 47, 55)
Neutrophils	Recruited to aged/fibrotic liver	↑ELANE, MPO, AZU1 (NETosis); immature neutrophils in advanced MASH	NETosis→HSC activation; ROS → paracrine senescence; NET-driven portal thrombosis	(5, 56–58)
B cells/TLS	Periportal TLS (around arteries)	GL7^+^IgD^+^ GC B cells; CXCL13^+^ FDC networks	TLS formation (NOTCH-deficiency); mature TLS → cirrhosis (PBC); FTY720 inhibits TLS	(59, 60)
Dendritic cells	Periportal/perivenous	↓MHC-II, CD80/86, IL-12; ↑PD-L1 (cirrhosis)	Impaired T-cell priming; Treg polarization; monocyte-DC hyperactivation in PSC	(17, 62)

KC, Kupffer cell; LAM/SAM, lipid/scar-associated macrophage; LSEC, liver sinusoidal endothelial cell; HSC, hepatic stellate cell; TRM, tissue-resident memory T cell; MAIT, mucosal-associated invariant T cell; NK, natural killer; TLS, tertiary lymphoid structure; DC, dendritic cell.

## 5 Senescence-driven remodeling of intercellular communication networks

### 5.1 SASP-mediated paracrine senescence propagation

SASP is the dominant mechanism reshaping intercellular communication in the aging liver. SASP effects are distance-dependent—paracrine senescence is most potent within a limited radius whose extent depends on local flow conditions and matrix interactions—a parameter that has not been precisely measured in the human hepatic lobule (10).

### 5.2 Extracellular vesicles as vectors of senescence signaling

EVs—including exosomes (30–150 nm), microvesicles (100–1,000 nm), and apoptotic bodies (1–5 μm)—are increasingly recognized as critical carriers of senescence-associated signals in the liver (63). Lipotoxic hepatocytes release EVs enriched in specific miRNAs (miR-423-5p, miR-146a-5p), oxidized lipids, and damage-associated proteins that are taken up by KCs and HSCs, promoting their inflammatory activation and senescence propagation (63).

### 5.3 Metabolic-immune crosstalk and Immunometabolic reprogramming

Metabolic reprogramming in the aging liver profoundly shapes immune cell communication. Aged KCs shift from OXPHOS toward glycolysis, leading to increased lactate production.

### 5.4 Chemokine network reprogramming

Chemokine network reprogramming is a central mechanism of spatial niche remodeling in the aging liver. Several chemokine axes undergo age-related dysregulation with distinct spatial consequences (4, 5, 26, 27, 31): CXCL2–CXCR2 axis: Cxcl2 + senescence-like macrophages in aged livers recruit neutrophils via this axis, creating focal inflammatory lesions (5).

CCL2–CCR2 axis: CCL2 released by senescent LSECs and KCs recruits Ly6C^hi^ inflammatory monocytes, which differentiate into MoMFs that progressively replace the embryonic KC pool (4, 22, 27).

CXCR4–CXCL12 axis: C-kit-deficient LSECs upregulate CXCR4, disrupting the local chemokine gradient and immune cell distribution (26).

CCL5–CCR5 axis: The intrahepatic microbiome–NKT–CCL5 signaling axis changes dynamically with age, influencing the overall size and activation state of the hepatic leukocyte pool (31).

CXCL13–CXCR5 axis: In TLS-forming regions, CXCL13 produced by FDCs and T follicular helper cells drives B-cell recruitment and lymphoid follicle organization (59, 60) (Table 3).

**Table 3:** Chemokine/receptor signaling axes in senescence-driven spatial niche remodeling.

Axis	Source	Target	Spatial context	Consequence	Ref.
CXCL2–CXCR2	Cxcl2^+^ senescence-like KCs	Neutrophils	Aged parenchyma	Neutrophil recruitment→NETosis→HSC activation→age-related injury	(5)
CCL2–CCR2	Senescent LSECs, KCs	Ly6C^hi^ monocytes	Portal→central gradient	MoMF recruitment→embryonic KC replacement→loss of homeostatic functions	(4, 22, 27)
CXCR4–CXCL12	C-kit-deficient LSECs (zone 3)	Mφ, neutrophils	Pericentral zone 3	Aberrant immune recruitment→pro-steatotic niche→MASH; blocked by AMD3100	(26)
CCL5–CCR5	NKT cells (microbiome-driven)	Leukocytes	Sinusoidal, age-dynamic	~90% hepatic leukocyte expansion; TRM-mediated HSC apoptosis	(31, 49)
CXCL13–CXCR5	FDCs, Tfh in TLS	B cells	Portal TLS areas	B-cell recruitment→lymphoid organization→TLS maturation→periportal fibrosis	(59, 60)
S1P–S1PR2	Activated platelets, damaged cells	Neutrophils, HSCs	Fibrotic septa	NET formation promotion; HSC fibrogenesis amplification	(56)

## 6 Pathogenic roles in chronic liver disease

### 6.1 Metabolic dysfunction-associated steatosis

Hepatic steatosis, the initial stage of MASLD, is characterized by excessive triglyceride accumulation in hepatocytes (>5% of hepatocytes containing lipid droplets). While traditionally viewed as benign, steatosis creates a pro-inflammatory metabolic milieu that primes the hepatic immune system for subsequent injury. In this stage, lipid-laden hepatocytes release lipotoxic mediators (free fatty acids, ceramides, oxidized phospholipids) that activate resident immune cells through pattern recognition receptors, initiating low-grade sterile inflammation. Key early events include modified KC lipid handling with impaired efferocytosis, LSEC metabolic stress in the pericentral zone, and altered hepatocyte-KC metabolic crosstalk that disrupts the homeostatic lipid-sensing functions of the hepatic immune niche. Although cellular senescence is not yet widespread at this stage, the metabolic perturbations of steatosis establish the conditions under which subsequent senescence induction occurs.

Chronic liver disease represents a progressive spectrum—steatosis → steatohepatitis → fibrosis → cirrhosis → hepatocellular carcinoma (HCC)—in which senescence-associated immune niche remodeling plays distinct, stage-specific pathogenic roles. MASLD/MASH is the most prevalent chronic liver disease globally, affecting approximately 25–30% of the world population, and is a leading cause of cirrhosis and HCC. Aging is among the strongest independent risk factors for MASLD progression to MASH and cirrhosis—MASH prevalence and fibrosis progression rate in patients over 60 years are 2- to 4-fold higher than in those under 40 (22, 64, 65). Below, we examine the contribution of senescent immune cells at each disease stage, progressing from early steatosis through end-stage liver disease.

**Figure 2:**
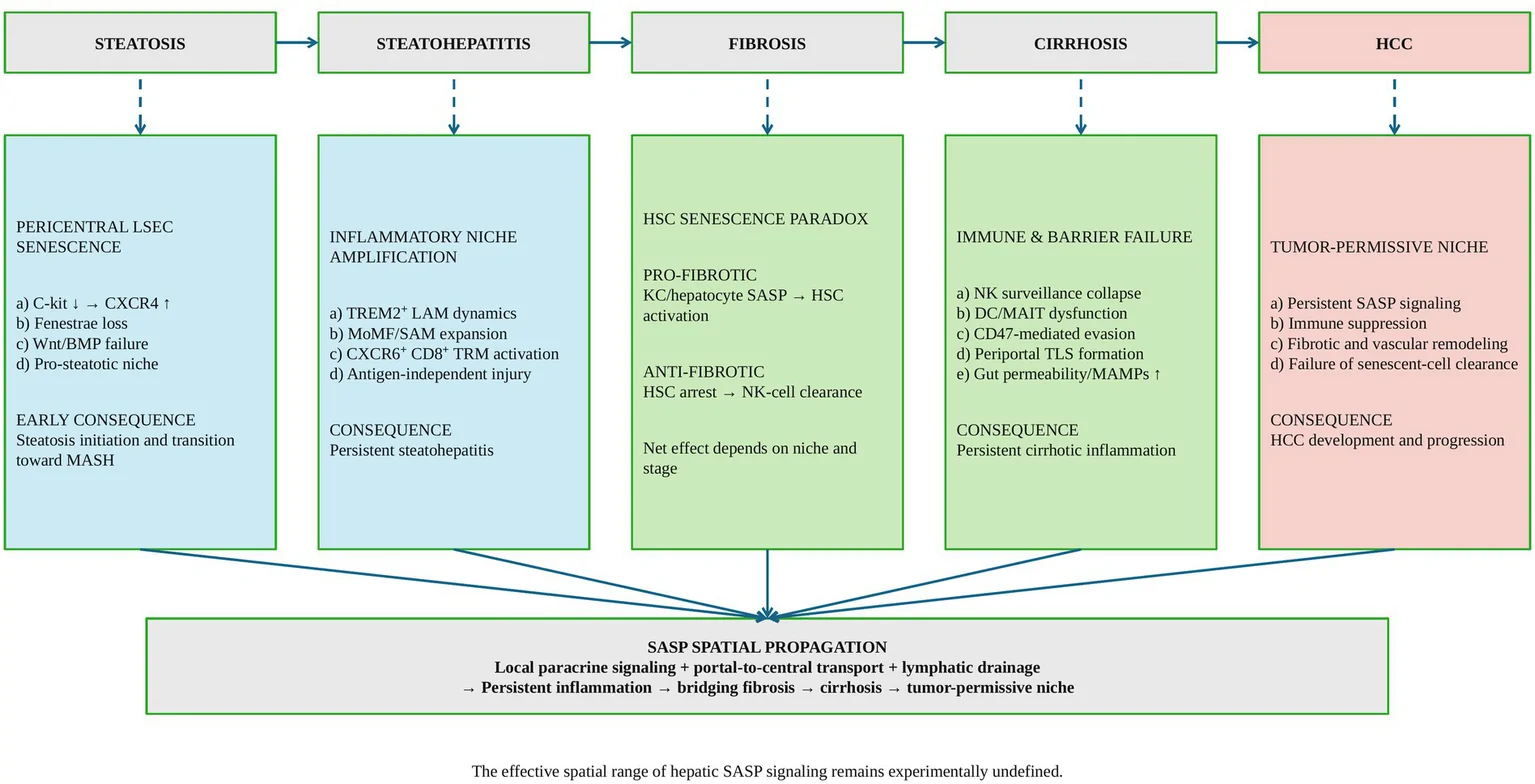
Spatial niche remodeling across the five stages of liver disease. The figure illustrates progression from steatosis through steatohepatitis, fibrosis, and cirrhosis to hepatocellular carcinoma (HCC). During steatosis, pericentral LSEC senescence, C-kit loss with CXCR4 upregulation, fenestrae loss, and Wnt/BMP failure establish a pro-steatotic niche and promote transition toward MASH. During steatohepatitis, TREM2 + lipid-associated macrophage dynamics, monocyte-derived and scar-associated macrophage expansion, CXCR6 + CD8 + TRM-cell activation, and antigen-independent injury amplify inflammation. During fibrosis, HSC senescence may promote fibrosis through KC- and hepatocyte-derived SASP signaling and HSC activation, or limit fibrosis through HSC arrest and NK-cell-mediated clearance, with the net effect depending on niche and disease stage. Cirrhosis is characterized by impaired NK-cell surveillance, dendritic-cell and MAIT-cell dysfunction, CD47-mediated immune evasion, periportal TLS formation, and increased gut permeability and MAMP exposure. In HCC, persistent SASP signaling, immune suppression, fibrotic and vascular remodeling, and defective senescent-cell clearance generate a tumor-permissive niche. SASP signals may propagate through local paracrine signaling, portal-to-central transport, and lymphatic drainage, although their effective spatial range remains experimentally undefined.

The stage-specific contributions of senescent immune cells to the full spectrum of chronic liver disease—from steatosis through steatohepatitis, fibrosis, and cirrhosis to HCC—are illustrated in Figure 2.

### 6.2 Steatohepatitis (MASH)

Senescent immune cells contribute to MASH pathogenesis through multiple interconnected mechanisms spanning the disease spectrum:

(1) LSEC senescence as a MASH initiator—the C-kit–CXCR4 axis identified by Duan et al. (26) triggers aberrant LSEC–macrophage–neutrophil crosstalk that establishes a pro-steatotic, pro-inflammatory niche in zone 3, initiating and perpetuating MASH pathology.
(2) The TREM2 + macrophage–ADAM17 antagonism. In compensated MASH, TREM2 + LAMs provide protective functions through efferocytosis of apoptotic lipotoxic hepatocytes and MMP-mediated fibrosis limitation (37, 38).
(3) Auto-aggressive CXCR6 + CD8 + TRM cells. The seminal study by Dudek et al. (66) identified auto-aggressive CXCR6 + CD8 + TRM cells that kill hepatocytes in an MHC-I-dependent but FasL-independent, antigen-independent manner, driven by IL-15 and acetate-mediated metabolic sensing—defining a new paradigm of “auto-aggression” in metabolic liver disease.
(4) Persistent NLRP3 inflammasome activation. The SIRT1–NLRP3 axis elucidated by Adjei-Mosi et al. (35) demonstrates that age-dependent loss of hepatic SIRT1 unleashes NLRP3 inflammasome activity in KCs, driving sustained IL-1*β* and HMGB1 release that perpetuates steatohepatitis and impairs fibrosis resolution.
(5) Senescent hepatocyte SASP propagation. Lipid toxicity-induced hepatocyte senescence triggers SASP secretion (IL-6, IL-8, CCL2, TGF-β) that paracrine-activates KCs and HSCs, accelerating steatohepatitis–fibrosis progression (10, 44).
(6) NETosis in steatohepatitis and fibrosis. Fa et al. (56) comprehensively documented the pro-inflammatory and pro-fibrotic roles of NETosis at each stage of NAFLD/NASH.

### 6.3 Liver fibrosis

As steatohepatitis persists, the cumulative burden of senescent cell accumulation, chronic SASP exposure, and progressive immune dysfunction converges on the fibrosis stage. Liver fibrosis is the final common pathway of chronic liver injury, and cirrhosis represents its end-stage with architectural distortion and functional decompensation. The transition from steatohepatitis to significant fibrosis represents the critical prognostic inflection point in chronic liver disease—it is the stage at which intervention may still reverse pathology and prevent progression to cirrhosis and HCC. At the fibrosis stage, senescence-associated immune niche remodeling operates through three sequentially considered dimensions:

(1) Formation and reinforcement of pro-fibrotic immune niches. Senescent KCs (M1-like polarization, NLRP3 activation) and capillarized LSECs (TGF-*β*, PDGF, Endothelin-1) create a permissive microenvironment for HSC trans-differentiation (5, 25, 26, 35). NETosis-derived factors (MPO, H3Cit, NE) directly activate HSCs and promote collagen deposition (56). MoMFs recruited via CCL2-CCR2 progressively replace the embryonic KC pool, losing homeostatic phagocytic and tolerogenic functions (4, 22).
(2) Progressive decline of anti-fibrotic immune surveillance. Aging- and fibrosis-associated impairment of NK-cell function reduces the clearance of activated or senescent HSCs. TGF-*β*-mediated downregulation of NKG2D and 2B4, reduced EP3–Itga4-dependent NK–HSC adhesion, HSC emperipolesis, and CD47-mediated evasion collectively promote the persistence of the fibrotic niche (46, 47, 67).
(3) The HSC senescence paradox and spatial heterogeneity. The net effect of HSC senescence on fibrosis depends on SASP quality and spatial context—periportal senescent HSCs, exposed to continuous gut microbial stimulation, may maintain a pro-inflammatory SASP that sustains fibrogenesis, while pericentral senescent HSCs may adopt a more favorable low-inflammatory SASP profile amenable to NK-mediated clearance (10, 41, 45). In human chronic liver disease, the balance favors the pro-fibrotic face, as activated myofibroblasts vastly outnumber p53/p21-positive senescent HSCs in cirrhotic livers. ASCT2 inhibition represents a proof-of-concept strategy to induce HSC senescence while restricting the pro-inflammatory SASP through interference with the IL-1α/NF-κB feedback loop (45).

The demonstration by Adjei-Mosi et al. (35) that age-dependent SIRT1 loss drives persistent NLRP3 activation and fibrosis represents a key mechanistic link between epigenetic aging and the failure of fibrosis resolution in elderly individuals (Table 4).

**Table 4:** Multi-dimensional mechanisms of senescence-driven liver fibrosis/cirrhosis: a spatial perspective.

Dimension	Key mechanism	Cellular mediators	Spatial pattern	Ref.
Pro-fibrotic niche	M1-KCs (↑IL-1β, TNF-α, CXCL1/2); capillarized LSECs (↓NO,↑TGF-β); NLRP3–IL-1β–HMGB1; NETosis→HSC activation	Aged KCs, LSECs, neutrophils, MoMFs	Portal-zone early fibrosis; zone 3 LSEC senescence→steatohepatitis	(5, 25, 26, 35, 56)
Immune surveillance decline	TGF-β↓NKG2D/2B4; ↓EP3 → ↓Itga4 → ↓NK-HSC adhesion; HSC emperipolesis; CD47↑ → macrophage evasion	NK cells, CD8^+^ TRM, macrophages	Septal edges (immune-active); septal cores (immune-privileged)	(46, 47, 67)
HSC senescence paradox	Pro-fibrotic: hepatocyte/KC SASP→HSC activation. Anti-fibrotic: HSC senescence→arrest+NK clearance. Net effect = f(SASP quality)	HSCs, NK cells	Periportal HSCs: pro-inflammatory SASP; pericentral: favorable SASP	(10, 41, 45)
Gut–liver barrier failure	Aged dysbiosis→↑permeability→portal LPS/bacterial DNA → TLR4/NLRP3 → endotoxin tolerance paradox	KCs, LSECs, MAIT, CD14^+^CD8^+^ TRM	Portal zone (highest MAMP); systemic spillover in decompensation	(28–30, 50)
TLS-associated fibrosis	NOTCH deficiency→spontaneous TLS; mature TLS (GC B cells, FDC, HEV) → local fibrosis	B cells, Tfh, FDCs, HEVs	Periportal; around parenchymal arteries	(59, 60)
Epigenetic aging→fibrosis persistence	Age-dependent SIRT1 loss→NLRP3 → IL-1β/HMGB1 → HSC persistence; DNAm age acceleration (PhenoAge, DunedinPACE)	Hepatocytes→KCs, global immune	Systemic (epigenetic clocks); portal→central NLRP3 gradient	(23, 35, 69)

KC, Kupffer cell; LSEC, liver sinusoidal endothelial cell; MoMF, monocyte-derived macrophage; NKT, natural killer T cell; FDC, follicular dendritic cell; Tfh, T follicular helper; TLS, tertiary lymphoid structure; MASH, metabolic dysfunction-associated steatohepatitis.

### 6.4 Cirrhosis

Cirrhosis represents the end-stage of chronic liver disease, characterized by diffuse bridging fibrosis, architectural distortion with regenerative nodule formation, and portal hypertension. At this stage, the cumulative effects of SASP-driven chronic inflammation, progressive immune exhaustion, and intestinal barrier failure converge to produce a profoundly immunocompromised hepatic microenvironment.

Gut bacterial translocation and the cirrhotic immune paradox. Breakdown of the intestinal barrier in cirrhosis leads to persistent portal LPS and bacterial DNA delivery, generating the endotoxin tolerance paradox in KCs and monocytes—blunted acute LPS responses with maintained chronic low-grade cytokine secretion. This is accompanied by functional exhaustion of MAIT and TRM cells (29, 30, 52, 53). In decompensated cirrhosis, systemic spillover of microbial products drives extrahepatic organ dysfunction and defines the transition from compensated to decompensated disease.

Tertiary lymphoid structures and B-cell immunity. NOTCH deficiency-induced hepatic TLS formation is linked to focal fibrosis (59); mature TLS in PBC correlate with higher cirrhosis prevalence (60); the contribution of TLS to metabolic and viral cirrhosis remains underexplored but may represent an important pathogenic mechanism. In the cirrhotic liver, persistent antigenic stimulation from gut microbial translocation, chronic SASP signals (IL-6, CCL chemokines), and impaired clearance of apoptotic debris may create conditions permissive for TLS maintenance and maturation.

In decompensated cirrhosis, the loss of intestinal barrier integrity combined with impaired hepatic reticuloendothelial function permits systemic spillover of microbial products (LPS, bacterial DNA) and pro-inflammatory mediators into the systemic circulation, driving extrahepatic organ dysfunction, acute-on-chronic liver failure, and immune paresis that underlies the high infection risk in this population (30, 61).

### 6.5 Hepatocellular carcinoma

Cirrhosis is the single strongest risk factor for HCC, and approximately 80–90% of HCCs arise in the context of liver fibrosis/cirrhosis, positioning senescence-associated immune niche remodeling as a permissive condition for hepatocarcinogenesis. The transition from cirrhosis to HCC represents the end-stage of the disease spectrum, where the cumulative effects of decades-long SASP-driven inflammation, impaired immune surveillance, and accumulated genomic alterations in chronically regenerating hepatocytes culminate in malignant transformation. In this section, we examine how senescence-associated immune dysfunction contributes specifically to HCC initiation, progression, and response to therapy.

Global decline of immune surveillance. Reduced phagocytosis and antigen presentation by senescent KCs, impaired immune cell homing due to LSEC capillarization, and multi-factorial suppression of NK and CD8 + TRM cell cytotoxicity (TGF-*β*, IL-10, lactate) collectively diminish immune surveillance against transformed hepatocytes (5, 22, 46, 52).

Epigenetic aging and anti-tumor immunity. HCC tumor tissue exhibits accelerated epigenetic aging (PhenoAge clock), which is strongly negatively correlated with NK cell infiltration (23).

The senescence–HCC carcinogenic paradox. Although cellular senescence is fundamentally a tumor-suppressive mechanism (preventing proliferation of DNA-damaged cells), the chronic SASP-driven inflammatory microenvironment paradoxically promotes carcinogenesis (10, 19).

Age-related differences in immunotherapy responses. Immune checkpoint inhibitors (ICIs, anti-PD-1/PD-L1, anti-CTLA-4) have revolutionized HCC systemic therapy.

## 7 Therapeutic strategies targeting senescent liver immune niches

### 7.1 Senolytic therapies: selective elimination of senescent cells

Senolytics are drugs that selectively eliminate senescent cells, aiming to restore the balance between senescence induction and senescent cell clearance. Despite promising preclinical data, several fundamental barriers have slowed the clinical translation of senotherapies for chronic liver disease. First, the lack of universal, specific senescent cell surface markers makes it difficult to confirm target engagement and on-target senolytic activity in human liver tissue—current senolytic trials rely on indirect biomarkers (circulating SASP factors, epigenetic clocks) rather than direct demonstration of senescent cell elimination. Second, the functional heterogeneity of senescence across cell types and disease stages means that pan-senolytic approaches risk eliminating beneficially senescent cells (e.g., senescent HSCs in fibrosis resolution, senescent hepatocytes with tumor-suppressive arrest) alongside pathologically senescent cells. Third, the liver’s unique architecture and dual blood supply pose pharmacokinetic challenges—first-pass metabolism may inactivate orally administered senolytics before they reach therapeutic concentrations in the hepatic sinusoid, and senescent cells in zone 3 (the most hypoxic region) may be less accessible to blood-borne drugs. Fourth, chronic liver disease patients often present with thrombocytopenia, portal hypertension, and impaired drug metabolism, contraindicating senolytics with significant toxicity profiles. Finally, the optimal timing, duration, and frequency of senolytic treatment (“hit-and-run” intermittent dosing vs. continuous senomorphic suppression) have not been established for liver-specific indications. These barriers are discussed in the context of specific agents below.

**Figure 3:**
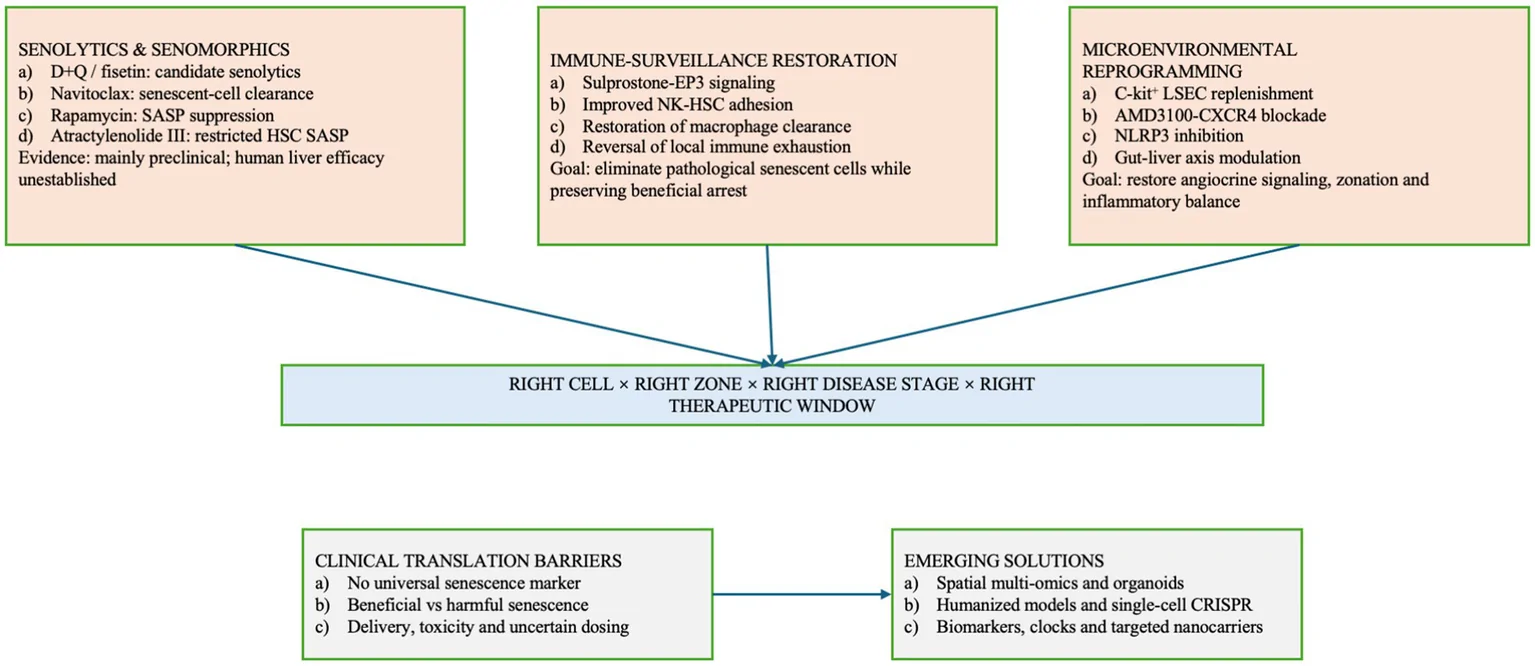
Spatial senotherapy strategies and barriers to clinical translation. Three complementary therapeutic approaches are shown. First, senolytic and senomorphic strategies include dasatinib plus quercetin and fisetin as candidate senolytics, navitoclax-mediated senescent-cell clearance, rapamycin-mediated SASP suppression, and atractylenolide III-mediated restriction of HSC-associated SASP. Second, immune-surveillance restoration includes enhancement of sulprostone-EP3 signaling, improvement of NK-cell-HSC adhesion, restoration of macrophage-mediated clearance, and reversal of local immune exhaustion, with the goal of eliminating pathological senescent cells while preserving beneficial cell-cycle arrest. Third, microenvironmental reprogramming includes C-kit+ LSEC replenishment, AMD3100-mediated CXCR4 blockade, NLRP3 inhibition, and gut-liver axis modulation to restore angiocrine signaling, zonation, and inflammatory balance. Successful therapy requires selection of the right cell, anatomical zone, disease stage, and therapeutic window. Major translational barriers include the absence of a universal senescence marker, difficulty distinguishing beneficial from harmful senescence, and unresolved delivery, toxicity, and dosing issues. Emerging solutions include spatial multi-omics and organoids, humanized models and single-cell CRISPR, and the development of biomarkers, aging clocks, and targeted nanocarriers.

Figure 3 provides an overview of spatially informed senotherapeutic strategies and the principal barriers to their clinical translation.

Dasatinib + Quercetin (D + Q): Dasatinib (a tyrosine kinase inhibitor) targets senescent adipocyte progenitors and endothelial cells; quercetin (a flavonoid inhibiting BCL-2/BCL-xL and PI3K/AKT signaling) targets senescent endothelial cells and HSCs (10, 41). The combination is currently being evaluated in a Phase I/II clinical trial for fibrotic NAFLD (NCT05506488; completed in February 2026 with 30 participants; results have not yet been posted or published), representing the most clinically advanced senolytic strategy for chronic liver disease. However, no efficacy data have been published to date.

Navitoclax (ABT-263): A BCL-2/BCL-xL/BCL-w family inhibitor that effectively eliminates senescent HSCs and partially depletes senescent KCs (10). However, thrombocytopenia (due to BCL-xL dependence of platelets) limits its long-term use.

Fisetin: A natural flavonoid that clears senescent cells through PI3K/AKT/mTOR pathway inhibition, with activity against senescent KCs and endothelial cells. Clinical trials for fisetin in age-related diseases are underway, though liver-specific trials have not been widely initiated (10).

C-kit+ LSEC infusion strategy: Duan et al. (26) introduced a novel “cellular replenishment” approach—infusion of healthy C-kit-expressing LSECs into aged or MASH mice to restore the depleted LSEC pool (Table 5).

**Table 5:** Senolytic, senomorphic, and microenvironmental reprogramming agents for aging-associated chronic liver disease.

Agent	Class	Target/mechanism	Disease model	Evidence level	Clinical status	Major limitation	Ref.
Dasatinib + Quercetin (D + Q)	Senolytic	Tyrosine kinase + BCL-2/PI3K/AKT inhibition; targets SnC HSC, LSEC	CCl4 + HFD mouse; NAFLD patient biopsies	Phase I/II RCT	NCT05506488: completed Feb 2026 (n = 30); results not published	No efficacy data available; histological endpoint pending; long-term safety unknown	(10, 41)
Navitoclax (ABT-263)	Senolytic	BCL-2/BCL-xL/BCL-w inhibitor → apoptosis; targets SnC HSC	CCl4 mouse fibrosis; radiation-induced SnC models	Preclinical (*in vivo*)	Not in clinical trial for liver disease	Thrombocytopenia (BCL-xL-dependent platelet survival); contraindicated in cirrhosis	(10)
Fisetin	Senolytic (flavonoid)	PI3K/AKT/mTOR inhibition → SnC clearance; targets SnC KC, LSEC	Mouse aging models; no liver-specific models	Preclinical (in vivo)	Phase II for age-related diseases; no liver-specific trial	Poor oral bioavailability; no liver-targeted formulation; broad mechanism limits specificity	(10)
Rapamycin	Senomorphic (mTORC1 inhibitor)	mTORC1 inhibition → SASP factor translation↓; targets KC, HSC, hepatocyte	Mouse lifespan extension studies; low-dose liver protocols	Preclinical (in vivo)	Lifespan extension in mice; liver-specific protocols under investigation	Broad immunosuppression risk; metabolic side effects with chronic use	(10)
C-kit+ LSEC infusion	Cellular replenishment	Restores C-kit angiocrine signaling; represses CXCR4 via CEBPA	Aged MASH-diet mouse; C-kit-KO mouse	Preclinical (in vivo)	Not in clinical trial	Cell therapy scalability and sourcing challenges; mouse-to-human LSEC functional differences	(26)
MCC950 (CRID3)	NLRP3 inhibitor	Selective NLRP3 inhibition → blocks IL-1β, HMGB1; targets aged KC, MoMF	Aged mouse CCl4 fibrosis	Preclinical (in vivo)	Not registered for liver indication	Short treatment window; NLRP3 role in host defense raises infection risk	(35)
Atractylenolide III	ASCT2 inhibitor (precision SASP)	ASCT2 inhibition → HSC senescence + restricted SASP via IL-1α/NF-κB	CCl4 mouse fibrosis	Preclinical (in vivo)	Not registered	Oral bioavailability concerns; off-target metabolic effects; PK/PD optimization needed	(45)
Sulprostone	EP3 agonist (immune restoration)	EP3 → PKC → Spic → Itga4↑ → NK-HSC adhesion↑; targets NK cells	CCl4 + BDL mouse fibrosis	Preclinical (*in vivo*)	Not registered	No EP3 agonist approved for liver; long-term safety unknown; efficacy in advanced fibrosis untested	(47)
AMD3100 (Plerixafor)	CXCR4 antagonist (microenvironment)	CXCR4 blockade → disrupts LSEC–Mφ–neutrophil crosstalk	Aged MASH-diet mouse	Preclinical (in vivo)	FDA-approved for HSC mobilization; MASH use preclinical only	Not liver-targeted; off-target stem cell mobilization; insufficient as monotherapy for established fibrosis	(26)

HSC, hepatic stellate cell; LSEC, liver sinusoidal endothelial cell; KC, Kupffer cell; MoMF, monocyte-derived macrophage; NK, natural killer; MASH, metabolic dysfunction-associated steatohepatitis; FDA, U. S. Food and Drug Administration; RCT, randomized controlled trial.

### 7.2 Senomorphic therapies: SASP modulation

Rather than eliminating senescent cells, senomorphic approaches aim to reprogram the SASP from a pro-inflammatory to a low-inflammatory or anti-inflammatory spectrum, preserving potentially beneficial functions of senescence (e.g., in wound healing and fibrosis resolution).

NF-κB pathway inhibition: IKK inhibitors (e.g., BMS-345541) and IκBα super-repressors can suppress the pro-inflammatory SASP (10, 41). However, systemic NF-κB inhibition may compromise normal immune responses.

mTOR signaling inhibition: Rapamycin and rapalogs reduce SASP factor translation and secretion via mTORC1 inhibition. Low-dose rapamycin extends lifespan and ameliorates age-related tissue phenotypes in mice, though specific effects on the liver immune niche require further study (10).

JAK/STAT pathway inhibition: JAK1/2 inhibitors (e.g., ruxolitinib) can attenuate IL-6/STAT3-driven SASP amplification loops (10). AG490 (a JAK inhibitor) pretreatment reduced senescence of transplanted mesenchymal stem cells in fibrotic livers and improved anti-fibrotic efficacy (2023).

SIRT1 activators: Adjei-Mosi et al. (35) demonstrated that SIRT1 activation restrains NLRP3 inflammasome overactivation and facilitates fibrosis resolution.

ASCT2 inhibition—precision SASP modulation: ASCT2 inhibition simultaneously induces HSC senescence and reprograms the SASP toward a low-inflammatory profile by impairing the IL-1α/NF-κB feedback loop (45). Atractylenolide III is a proof-of-concept ASCT2 inhibitor with *in vivo* anti-fibrotic efficacy (45).

### 7.3 Immune surveillance restoration

Restoring and enhancing the capacity of the immune system to clear senescent cells is another important therapeutic direction.

NK cell functional enhancement: Tao et al. (47) demonstrated that pharmacological EP3 activation (sulprostone) enhances NK cell adhesion to and killing of activated HSCs, alleviating liver fibrosis.

TGF-*β* signaling blockade: TGF-β is the dominant immunosuppressive factor in advanced fibrosis and cirrhosis that inhibits NK cell and CD8 + T-cell function. TGF-β receptor inhibitors (galunisertib) or neutralizing antibodies, combined with NK cell activation, may overcome the immunosuppressive barrier in cirrhosis (46).

CD47–SIRPα axis blockade: Senescent HSCs upregulate CD47 to inhibit macrophage efferocytosis. Anti-CD47 antibodies or SIRPα-Fc fusion proteins may restore macrophage-mediated clearance of senescent HSCs (10, 67).

CAR-NK/CAR-T cell approaches: Chimeric antigen receptor (CAR)-engineered immune cells targeting senescence-specific surface markers (uPAR, DPP4, NKG2D ligands) may serve as precision senolytic tools, although liver-directed CAR approaches remain at the preclinical stage.

### 7.4 Metabolic and microenvironmental reprogramming

CXCR4 blockade: Duan et al. (26) demonstrated that blocking CXCR4 (with AMD3100/Plerixafor) abolishes aberrant LSEC–macrophage–neutrophil crosstalk.

CXCL2–CXCR2 axis blockade: Liu et al. (5) showed that targeting CXCL2–CXCR2 attenuates neutrophil recruitment and NETosis-driven liver injury in aged mice.

NLRP3 inflammasome inhibition: Adjei-Mosi et al. (35) demonstrated that MCC950 alleviates liver fibrosis in aged mice.

### 7.5 Gut microbiota modulation

Probiotics/prebiotics/synbiotics: Adhikary et al. (29) reviewed how gut microbiota manipulation can intervene in age-related chronic liver disease.

Fecal microbiota transplantation (FMT): Preliminary studies of FMT in cirrhosis have shown benefits in reducing endotoxemia and improving hepatic encephalopathy, but data in aging-related liver disease are lacking (28, 29).

FTY720 (fingolimod) and TLS inhibition: FTY720 inhibited TLS formation and relieved cholangitis and fibrosis in a PBC mouse model (60). As an approved drug for multiple sclerosis, its repurposing for autoimmune liver diseases with prominent TLS involvement warrants investigation.

### 7.6 Epigenetic reprogramming and hepatic rejuvenation

Epigenetic reprogramming: The 2023 finding that liver regeneration partially reverses aberrant H3K27me3 patterns suggests that endogenous rejuvenation programs exist and can be therapeutically harnessed (68). Partial reprogramming using Yamanaka factors (Oct4, Sox2, Klf4, c-Myc) has shown anti-aging effects across multiple tissues, though liver-specific studies remain preclinical (68).

Telomerase gene therapy: Penrice et al. (19) reviewed the potential of telomerase gene therapy for telomere biology disorders with hepatic involvement.

Epigenetic clocks as pharmacodynamic biomarkers: Epigenetic aging clocks (PhenoAge, DunedinPACE, IMM-Age) can serve as integrated molecular readouts of biological age in clinical trials of senolytic/senomorphic therapies, enabling patient stratification and treatment response monitoring (23, 69).

## 8 Summary and future perspectives

### 8.1 Core scientific conclusions

This review has systematically examined the emerging evidence linking senescence-associated spatial niche remodeling of liver-resident immune cells to the pathogenesis and progression of chronic liver disease. The weight of current evidence supports three overarching conclusions. First, liver-resident immune cell senescence is not a uniform process but is spatially organized along the portal-to-central axis of the hepatic lobule, with distinct cell types exhibiting zone-specific susceptibility to senescence induction, functional decline, and SASP production. This spatial dimension fundamentally determines the tissue-level impact of immune senescence and distinguishes hepatic immunosenescence from that of other organs. Second, spatial niche remodeling—encompassing cell-intrinsic senescence, spatial redistribution of immune subsets, and communication network rewiring—represents the mechanistic link connecting molecular aging programs to organ-level pathology. The progressive replacement of embryonic KCs by monocyte-derived macrophages, the periportal-to-pericentral spread of HSC activation, the zone-specific vulnerability of LSECs to capillarization, and the formation of spatially restricted TLS all exemplify how senescence reshapes the hepatic immune landscape across multiple scales. Third, the global rewiring of intercellular communication networks—through SASP gradients, extracellular vesicles, metabolic crosstalk, and chemokine reprogramming—amplifies the spatial reach of senescent cell effects and locks the hepatic immune ecosystem into a self-perpetuating inflammatory state that underlies the progression from steatosis to steatohepatitis, fibrosis, cirrhosis, and ultimately hepatocellular carcinoma.

### 8.2 Critical unresolved questions

Despite the substantial progress reviewed here, several fundamental questions remain unresolved and represent high-priority directions for future investigation. At the molecular level, the precise mechanisms governing differential senescence susceptibility across lobular zones—why periportal KCs are more prone to inflammatory senescence while pericentral LSECs are more vulnerable to metabolic stress-induced senescence—remain poorly defined. The molecular basis of zone-specific senescence likely involves the integration of microenvironmental signals (oxygen tension, Wnt/*β*-catenin gradients, TLR ligand exposure, bile acid concentrations) with cell-type-specific chromatin landscapes, but direct experimental dissection of these interactions using spatially resolved multi-omics approaches has not been performed. At the cellular level, liver TRM cells, ILCs, and B cells represent the most critically understudied populations in the context of immune aging. These long-lived, tissue-resident lymphocyte populations are positioned to both sense and shape the aging hepatic microenvironment, yet systematic characterization of their age-related transcriptional, epigenetic, and functional changes is virtually absent from the literature. At the tissue level, the effective spatial range of SASP action in the human hepatic lobule, the role of the aging intrahepatic microbiome in shaping liver immunity, and the functional significance of TLS maturation in age-associated fibrosis all remain open questions requiring spatially resolved experimental approaches. At the translational level, whether epigenetic aging clocks (PhenoAge, DunedinPACE, IMM-Age) capture functional immune senescence in the liver, whether elderly HCC patients exhibit differential responses to immune checkpoint inhibitors, and whether sex-specific immune aging trajectories necessitate sex-stratified therapeutic strategies are questions of immediate clinical relevance that demand prospective, biomarker-integrated clinical studies (Table 6).

**Table 6:** Critical knowledge gaps and proposed future research directions in liver immune senescence.

Priority area	Knowledge gap	Proposed approach	Expected impact
TRM/ILC aging	No systematic studies of age-related changes in liver TRM or ILCs	Longitudinal scRNA-seq + TCR-seq of liver TRM; ILC fate-mapping in aged mice	Define mechanisms of age-associated decline in hepatic lymphocyte surveillance
Zone-specific senescence	Drivers of differential senescence susceptibility (portal vs. central) undefined	Spatial multi-omics (Visium HD + metabolomics + proteomics); zone-specific AAV perturbation	Enable zone-targeted anti-senescence therapies
SASP spatial range	Effective paracrine radius of SASP in hepatic lobule unmeasured	Spatial transcriptomics + cytokine capture; computational 3D lobular modeling	Define spatial “danger zone” around senescent cells
Intrahepatic microbiome aging	Composition, dynamics, functional significance in human aging unexplored	16S rRNA/spatial metagenomics of aged human liver; correlation with NKT/CCL5 function	Novel microbiome→NKT → immune landscape axis
B cell/TLS aging	Hepatic B cell immunosenescence almost completely unstudied	B cell scRNA-seq + BCR-seq of aged liver; spatial TLS mapping across age	Define humoral immunity role in age-associated fibrosis
Sex differences	Impact of sex hormones on liver immune senescence trajectories uncharacterized	Sex-stratified liver immune aging atlases; gonadectomy+HRT studies	Personalized anti-senescence strategies by sex
Epigenetic clocks vs. function	Causal link between accelerated clocks and functional senescence unclear	Multi-omics integration: DNAme+scRNA-seq + functional assays; longitudinal CLD cohorts	Validate clocks as pharmacodynamic biomarkers for senolytic trials
Age-stratified immunotherapy	Whether elderly HCC patients differ in ICI response/irAE unknown	Age-stratified re-analysis of HCC ICI trials; prospective immune monitoring	Optimize ICI selection for elderly HCC population
EV senescence signalome	How aging alters EV production, cargo, and function uncharacterized	Multi-omic EV profiling from young vs. aged liver cells; functional transfer assays	Identify senescence-specific EV cargo as biomarkers/therapeutic targets

TRM, tissue-resident memory T cell; ILC, innate lymphoid cell; EV, extracellular vesicle; NKT, natural killer T cell; TLS, tertiary lymphoid structure; ICI, immune checkpoint inhibitor; irAE, immune-related adverse event; CLD, chronic liver disease.

### 8.3 Future technological and research directions

Looking forward, we envision that the next 5 years will witness transformative advances in our understanding and therapeutic manipulation of liver immune senescence, driven by the convergence of several technological and conceptual developments. Cellular senescence is increasingly recognized as a heterogeneous biological state characterized by stable cell-cycle arrest, extensive phenotypic remodeling, and context-dependent secretory programs rather than a single uniform phenotype (71). In the liver, these senescence-associated changes may exert both protective and pathogenic effects depending on cell type, disease stage, and tissue context (73). The integration of spatial transcriptomics (Visium HD, MERFISH, Xenium), spatial proteomics (IMC, MIBI-TOF, CODEX), and spatial metabolomics will enable the construction of three-dimensional multi-omics atlases of the aging human liver, resolving—for the first time—the precise spatial relationships between immune cell identity, senescence status, molecular phenotype, and functional output. Such approaches will be particularly valuable for defining tissue-resident immune niches, including hepatic CD69+CD103+CD8+ resident memory T-cell populations that have already been implicated in immune-mediated liver disease (75), as well as neutrophil-associated inflammatory programs enriched within fibrotic liver microenvironments (76). Coupled with artificial intelligence and systems biology approaches—including graph neural networks that model cell–cell communication in spatial context and spatial variational autoencoders that reconstruct multi-scale tissue organization—these atlases will enable in silico perturbation screens predicting the effects of senolytic or senomorphic interventions on specific immune niches.

Technological convergence with artificial intelligence and systems biology: Computational modeling of liver immune senescence—from molecular interaction networks to tissue-scale spatial simulations—is poised to transform descriptive knowledge into predictive frameworks. Deep learning models trained on spatial multi-omics datasets can identify novel senescence regulators, predict cell–cell communication hubs, and simulate the effects of senotherapeutic perturbations across the hepatic lobule. Particular attention should be given to the senescence-associated secretory phenotype (SASP), which can propagate inflammatory and senescent signals to neighboring cells through complex cytokine- and inflammasome-regulated paracrine networks (72). These computational approaches will be particularly powerful when integrated with pooled single-cell CRISPR screens in hepatic organoid co-cultures and humanized mouse models, enabling systematic, high-throughput identification of genes and pathways that regulate immune cell senescence, SASP production, metabolic reprogramming, and spatial positioning in a human-relevant context. Studies of age- and metabolism-associated alterations in hepatic myeloid cells, including investigations of PKM2-dependent metabolic regulation in metabolic dysfunction-associated fatty liver disease, further illustrate the potential importance of immunometabolic pathways in liver aging and disease progression (77). The development of liver organoid systems incorporating multiple resident immune cell types and replicating lobular zonation (through microfluidic oxygen and morphogen gradients) represents a particularly promising platform for preclinical senotherapeutic testing that bridges the gap between murine models and human clinical trials.

For clinical translation, we advocate the incorporation of epigenetic aging clocks (PhenoAge, DunedinPACE, IMM-Age) and validated senescence-associated biomarkers (circulating SASP factors, sTREM2, NET markers, EV-encapsulated miRNAs) into the design of senolytic and senomorphic clinical trials for chronic liver disease (71, 73). Given that extracellular shedding of immune-regulatory surface molecules can substantially alter immune surveillance and generate potentially measurable circulating products, soluble ectodomain-derived molecules may also warrant exploration as complementary biomarkers in appropriate disease settings (74). These biomarkers would serve triple purposes: patient stratification (identifying individuals with accelerated hepatic immune aging who may benefit most from senotherapy), pharmacodynamic monitoring (confirming target engagement and biological effect), and personalized treatment optimization (determining dosing frequency and treatment duration). The establishment of longitudinal aging cohorts spanning the full spectrum from healthy aging through compensated and decompensated chronic liver disease, with deep multi-omic and imaging phenotyping at regular intervals, will be essential for resolving the dynamic trajectories of liver immune aging, identifying critical intervention windows, and validating surrogate endpoints for clinical trials. Finally, cross-species comparative analyses of liver immune aging—leveraging the divergent lifespans and liver disease susceptibilities of mouse, non-human primate, and human—may reveal conserved core aging mechanisms and species-specific vulnerabilities that refine therapeutic target selection and improve the predictive validity of preclinical models.

## Glossary

ACLF: acute-on-chronic liver failure
ADAM17: a disintegrin and metalloproteinase 17
AIH: autoimmune hepatitis
AKT: protein kinase B
ALT: alanine aminotransferase
ASCT2: alanine-serine-cysteine transporter 2
ATG5: autophagy related 5
AAV: adeno-associated virus
BCL-2: B-cell lymphoma 2
BCL-xL: B-cell lymphoma extra large
BCL-w: B-cell lymphoma like (W)
BMP: bone morphogenetic protein
BRD4: bromodomain containing 4
BCR: B cell receptor
CAR: chimeric antigen receptor
cDC1: conventional dendritic cell 1
cDC2: conventional dendritic cell 2
CD10: cluster of differentiation 10
CD107a: lysosomal associated membrane protein 1
CD14: cluster of differentiation 14
CD141: cluster of differentiation 141
CD163: cluster of differentiation 163
CD177: cluster of differentiation 177
CD1c: cluster of differentiation 1c
CD206: cluster of differentiation 206
CD32b: cluster of differentiation 32b
CD36: cluster of differentiation 36
CD38: cluster of differentiation 38
CD47: cluster of differentiation 47
CD69: cluster of differentiation 69
CD79B: cluster of differentiation 79B
CD80: cluster of differentiation 80
CD86: cluster of differentiation 86
CD103: cluster of differentiation 103
cGAS: cyclic GMP-AMP synthase
CCL2: C-C motif chemokine ligand 2
CCL5: C-C motif chemokine ligand 5
CCR2: C-C chemokine receptor 2
CCR5: C-C chemokine receptor 5
CEBPA: CCAAT enhancer binding protein alpha
Clec4F: C-type lectin domain family 4 member F
CLD: chronic liver disease
CODEX: co-detection by indexing
COL6A3: collagen type VI alpha 3 chain
CRID3: cytokine release inhibitory drug 3
CRISPR: clustered regularly interspaced short palindromic repeats
CXCL1: C-X-C motif chemokine ligand 1
CXCL2: C-X-C motif chemokine ligand 2
CXCL5: C-X-C motif chemokine ligand 5
CXCL8: C-X-C motif chemokine ligand 8
CXCL12: C-X-C motif chemokine ligand 12
CXCL13: C-X-C motif chemokine ligand 13
CXCR2: C-X-C chemokine receptor 2
CXCR3: C-X-C chemokine receptor 3
CXCR4: C-X-C chemokine receptor 4
CXCR5: C-X-C chemokine receptor 5
CXCR6: C-X-C chemokine receptor 6
cNK: conventional NK (cell)
eNOS: endothelial nitric oxide synthase
DAMP: damage-associated molecular pattern
DCs: dendritic cells
DDR: DNA damage response
DNA-SCARS: DNA segments with chromatin alterations reinforcing senescence
DNAm: DNA methylation
DPP4: dipeptidyl peptidase 4
DunedinPACE: Dunedin Pace of Aging Computed from the Epigenome
ECM: extracellular matrix
ELANE: elastase neutrophil expressed
EMP: erythro-myeloid progenitors
Eomes: eomesodermin
EP3: prostaglandin E2 receptor 3
EVs: extracellular vesicles
FABP5: fatty acid binding protein 5
FasL: Fas ligand
Fc: fragment crystallizable
FCGR3A: Fc fragment of IgG receptor IIIa
FDC: follicular dendritic cell
FMT: fecal microbiota transplantation
FTY720: fingolimod
GC: germinal center
GL7: lymphocyte antigen 77
GPNMB: glycoprotein non-metastatic melanoma protein B
HBV: hepatitis B virus
HCC: hepatocellular carcinoma
HCV: hepatitis C virus
HEVs: high endothelial venules
HFD: high-fat diet
HGF: hepatocyte growth factor
HMGB1: high mobility group box 1
Hmox1: heme oxygenase 1
HRT: hormone replacement therapy
HSCs: hepatic stellate cells
H3Cit: citrullinated histone H3
ICI: immune checkpoint inhibitor
IKK: IκB kinase
ILC1: innate lymphoid cell 1
ILC2: innate lymphoid cell 2
ILC3: innate lymphoid cell 3
ILCs: innate lymphoid cells
IL-1*β*: interleukin 1 beta
IL-6: interleukin 6
IL-8: interleukin 8
IL-10: interleukin 10
IL-12: interleukin 12
IL-13: interleukin 13
IL-17A: interleukin 17A
IL-22: interleukin 22
IL-33: interleukin 33
IMC: imaging mass cytometry
IMM-Age: Immune Age
IFN-*α*: interferon alpha
IFN-β: interferon beta
IFN-*γ*: interferon gamma
IgD: immunoglobulin D
IgG: immunoglobulin G
iNKT: invariant natural killer T
irAE: immune-related adverse event
Itga4: integrin alpha 4
JAK: Janus kinase
KC: Kupffer cell
LAMs: lipid-associated macrophages
LPS: lipopolysaccharide
LSECs: liver sinusoidal endothelial cells
LTBP4: latent transforming growth factor beta binding protein 4
LYVE1: lymphatic vessel endothelial hyaluronan receptor 1
MAIT: mucosal-associated invariant T (cells)
MAMP: microbe-associated molecular pattern
MARCO: macrophage receptor with collagenous structure
MASLD: metabolic dysfunction-associated steatotic liver disease
MHC-II: major histocompatibility complex class II
MHC: major histocompatibility complex
MASH: metabolic dysfunction-associated steatohepatitis
MICA: MHC class I polypeptide-related sequence A
MICB: MHC class I polypeptide-related sequence B
MIBI-TOF: multiplexed ion beam imaging by time of flight
miRNAs: microRNAs
miR-223: microRNA 223
MOMP: mitochondrial outer membrane permeabilization
MoMFs: monocyte-derived macrophages
MPO: myeloperoxidase
MR1: MHC class I related molecule 1
mTOR: mechanistic target of rapamycin
mTORC1: mTOR complex 1
MMP: matrix metalloproteinase
NAFLD: nonalcoholic fatty liver disease
NAMs: NASH-associated macrophages
NAS: NAFLD activity score
NASH: nonalcoholic steatohepatitis
NE: neutrophil elastase
NETosis: neutrophil extracellular trap formation
NF-κB: nuclear factor kappa B
NK: natural killer
NKG2D: natural killer group 2D
NKp30: natural cytotoxicity triggering receptor 3
NLRP3: NOD-like receptor family pyrin domain containing 3
NO: nitric oxide
NOTCH: neurogenic locus notch homolog protein
NKT: natural killer T (cells)
OXPHOS: oxidative phosphorylation
pDCs: plasmacytoid dendritic cells
PAMPs: pathogen-associated molecular patterns
PBC: primary biliary cholangitis
PD-1: programmed cell death protein 1
PD-L1: programmed death-ligand 1
PhenoAge: Phenotypic Age
RCT: randomized controlled trial
PI3K: phosphoinositide 3-kinase
PKC: protein kinase C
PSC: primary sclerosing cholangitis
Rac1: Ras-related C3 botulinum toxin substrate 1
Rbpj: recombination signal binding protein for immunoglobulin kappa J region
ROS: reactive oxygen species
Rspo3: R-spondin 3
SAMs: scar-associated macrophages
SASP: senescence-associated secretory phenotype
SCFAs: short-chain fatty acids
scRNA-seq: single-cell RNA sequencing
SIRT1: sirtuin 1
SnC: senescent cell
SIRPα: signal regulatory protein alpha
snRNA-seq: single-nucleus RNA sequencing
SOCS3: suppressor of cytokine signaling 3
Spic: spleen focus forming virus proviral integration oncogene
SPP1: secreted phosphoprotein 1
sTREM2: soluble TREM2
STING: stimulator of interferon genes
STAT: signal transducer and activator of transcription
STAT3: signal transducer and activator of transcription 3
STAT6: signal transducer and activator of transcription 6
TCR: T cell receptor
Tfh: T follicular helper
TGF-*β*: transforming growth factor beta
TIM4: T cell immunoglobulin and mucin domain containing 4
TIM-3: T cell immunoglobulin and mucin domain containing 3
TLR2: Toll-like receptor 2
TLR4: Toll-like receptor 4
TLS: tertiary lymphoid structures
TNF-*α*: tumor necrosis factor alpha
TRM: tissue-resident memory T (cells)
TREM2: triggering receptor expressed on myeloid cells 2
uPAR: urokinase plasminogen activator receptor
ULBPs: UL16 binding proteins
VCAM-1: vascular cell adhesion molecule 1
Wnt: Wingless-related integration site
XCR1: X-C motif chemokine receptor 1
2B4: CD244/natural killer cell receptor 2B4
16S rRNA: 16S ribosomal RNA
